# CCL14 is a prognostic biomarker and correlates with immune infiltrates in hepatocellular carcinoma

**DOI:** 10.18632/aging.102656

**Published:** 2020-01-12

**Authors:** Yurong Gu, Xiangyong Li, Yanhua Bi, Yubao Zheng, Jialiang Wang, Xiaoyan Li, Zexuan Huang, Lubiao Chen, Yanlin Huang, Yuehua Huang

**Affiliations:** 1 Department of Infectious Diseases, The Third Affiliated Hospital of Sun Yat-sen University, Guangzhou, China; 2 Guangdong Provincial Key Laboratory of Liver Disease Research, The Third Affiliated Hospital of Sun Yat-sen University, Guangzhou, China

**Keywords:** CCL14, hepatocellular carcinoma, biomarker, immunity, exhaustion marker

## Abstract

C-C motif chemokine ligand 14 (CCL14) is a chemokine promoting the activation of immune cells. However, the relationship between CCL14 expression, tumor immunity, and prognosis in Hepatocellular Carcinoma (HCC) remain unclear. CCL14 expression and its influence on tumor prognosis were analyzed by the ONCOMINE, Tumor Immune Estimation Resource (TIMER) and Kaplan-Meier plotter. The relationship between CCL14 expression and tumor immunity were analyzed by TIMER and Gene Expression Profiling Interactive Analysis (GEPIA). CCL14 expression was significantly lower in several human cancers, including HCC, than in corresponding normal tissues. CCL14 expression in HCC tissues correlated with prognosis. Low CCL14 expression associated with poorer overall survival, disease-specific survival, progression-free survival, and relapse-free survival in multiple cohorts of HCC patients, particularly at early disease stages (stage 1+2 or grade 2). CCL14 showed strong correlation with tumor-infiltrating B cells, CD4^+^ and CD8^+^ T cells, macrophages, neutrophils, and dendritic cells. CCL14 expression in HCC negatively correlated with expression of several immune cell markers, including exhausted T cell markers, PD-1, TIM-3 and CTLA-4, suggesting its role in regulating tumor immunity. These findings demonstrate that CCL14 is a potential prognostic biomarker that determines cancer progression and correlated with tumor immune cells infiltration in HCC.

## INTRODUCTION

Hepatocellular carcinoma (HCC) is the fifth most common tumor and the second most common cause of cancer-related deaths worldwide according to the World Health Organization [1]. The major risk factors for developing HCC include chronic hepatitis B or C virus infections, non-alcoholic steatohepatitis, and alcohol-related liver diseases [2, 3]. While approximately 20% HCC patients are diagnosed in the early stages and amenable for treatments by surgical therapies, liver transplantation and radiofrequency ablation, the remaining patients are diagnosed in advanced stages and are not eligible for the curative treatments [4–7]. Furthermore, a high rate of disease recurrence contributes to poor outcomes in majority of HCC patients [8, 9]. Therefore, a deeper understanding of the molecular mechanisms involved in HCC pathogenesis is critical for developing new treatments to improve survival rates.

Chronic liver inflammation causes repeated hepatocellular damage and regeneration, and promotes HCC initiation and growth [10]. Tumor-related chronic inflammation also involves impaired secretion of pro-inflammatory cytokines and recruitment of immune cells to tumor sites [10–12]. Chemokines are small secretory proteins that belong to C, CXC, CC or CX3C families based on the position of the first two cysteine residues [13, 14]. Chemokines play a critical role in the recruitment and activation of immune cells to the sites of injury and infection, and contribute to cancer progression, invasion and metastasis [15–18]. The CC chemokine ligand 14 (CCL14) is a CC-type chemokine that is constitutively expressed in several tissues [19]. The active form of CCL14 binds to CCR1, CCR3, and CCR5, and promotes chemotaxis of monocytes, eosinophils, and T lymphoblasts [13, 20]. Zhang et al. showed that CCL14, TMEM88, and CLEC3B are biomarkers that can predict the survival outcomes of HCC patients [21]. However, the precise function and mechanism of CCL14 in HCC progression is still unclear.

In this study, we comprehensively analyzed the expression of CCL14, its correlation with prognosis in different types of tumors including HCC, and the status of different tumor-infiltrating immune cells based on expression of specific markers using the ONCOMINE, Kaplan-Meier plotter, Gene Expression Profiling Interactive Analysis (GEPIA), and Tumor Immune Estimation Resource (TIMER) databases. Our results shed light on the important role of CCL14 in HCC prognosis and provided an underlying mechanism that CCL14 expression might modulate tumor immunity by regulating the infiltration of immune cells in HCC.

## RESULTS

### The levels of CCL14 mRNA in HCC and other cancers

**Figure 1:**
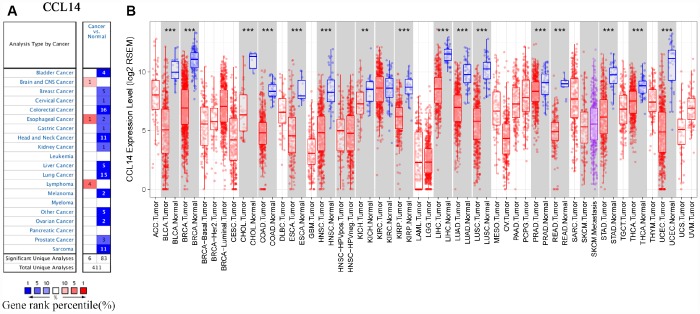
**CCL14 expression in different types of human cancers.** (**A**) High or low expression of CCL14 in different human cancer tissues compared with normal tissues using the Oncomine database. (**B**) The level of CCL14 expression in different tumor types from the TCGA database in TIMER. Note: *P < 0.05, **P < 0.01, ***P < 0.001.

Gene expression analyses using the ONCOMINE database showed that CCL14 mRNA levels were significantly lower in liver, bladder, breast, cervical, colorectal, gastric, head and neck, kidney, lung, ovarian prostate, and sarcoma cancer tissues, and significantly higher in the brain, esophageal, and lymphoma cancers compared with the corresponding normal tissues (Figure 1A and Supplementary Table 1). The analysis of TCGA RNA-seq data using the TIMER database showed that CCL14 mRNA expression was significantly lower in BLCA (bladder urothelial carcinoma), BRCA (breast invasive carcinoma), CHOL (cholangiocarcinoma), COAD (colon adenocarcinoma), ESCA (esophageal carcinoma), HNSC (head and neck cancer), KICH (kidney chromophobe), KIRP (kidney renal papillary carcinoma), HCC (liver hepatocellular carcinoma), LUAD (lung adenocarcinoma), LUSC (lung squamous cell carcinoma), PRAD (prostate adenocarcinoma), READ (rectum adenocarcinoma), STAD (stomach adenocarcinoma), THCA(thyroid carcinoma), and UCEC (uterine corpus endometrial carcinoma) tissues compared with the corresponding normal tissues (Figure 1B). None of the tumor tissues analyzed in the TIMER database showed higher CCL14 expression compared with the corresponding normal tissues. These data showed that CCL4 expression was deregulated in cancer tissues.

**Table 1:** Correlation of CCL14 mRNA expression and prognosis in hepatocellular carcinoma with different clinicopathological factors by Kaplan-Meier plotter.

**Clinicopathological factors**	**Overall survival**				**Progression-free survival**		
	**N**	**Hazard ratio**	**P-value**		**N**	**Hazard ratio**	**P-value**
SEX							
Female	118	0.59(0.34-1.03)	**0.061**		120	0.7(0.42-1.16)	0.16
Male	246	0.37(0.23-0.6)	**1.8e-05**		246	0.56(0.39-0.8)	**0.0013**
Stage							
1	170	0.45(0.24-0.86)	**0.014**		171	0.91(0.55-1.49)	0.69
2	83	0.39(0.17-0.9)	**0.023**		85	0.74(0.41-1.33)	0.31
1+2	253	0.52(0.32-0.85)	**0.0074**		256	0.64(0.44-0.93)	**0.019**
3	83	0.45(0.25-0.82)	**0.0075**		85	1(0.58-1.72)	1
4	5	-	**-**		5	-	-
3+4	87	0.5(0.28-0.89)	**0.016**		90	1.03(0.61-1.74)	0.92
Grade							
1	55	0.67(0.26-1.72)	0.4		55	0.89(0.4-1.95)	0.77
2	174	0.52(0.31-0.87)	**0.012**		175	0.57(0.37-0.88)	**0.01**
3	118	0.39(0.21-0.75)	**0.003**		119	0.74(0.45-1.21)	0.23
4	12	-	-		12	-	-
AJCC_T							
1	180	0.48(0.26-0.89)	**0.017**		180	0.86(0.53-1.39)	0.54
2	90	0.35(0.16-0.76)	**0.0059**		92	0.68(0.4-1.18)	0.17
3	78	0.47(0.26-0.86)	**0.012**		78	1(0.57-1.77)	0.99
4	13	-	-		13	-	-
Vascular invasion							
yes	90	0.46(0.21-1.02)	0.05		91	0.73(0.42-1.29)	0.28
None	203	0.46(0.27-0.78)	**0.0033**		204	0.68(0.44-1.06)	0.088
Race							
White	181	0.66(0.41-1.04)	0.071		183	0.62(0.41-0.92)	**0.016**
Asian	155	0.3(0.16-0.58)	**0.00015**		155	0.61(0.38-0.98)	**0.04**
Alcohol consumption							
yes	115	0.52(0.27-1.0)	**0.0048**		115	0.5(0.3-0.85)	**0.0088**
none	202	0.53(0.33-0.85)	**0.0068**		204	0.66(0.44-0.99)	**0.042**
Virus hepatitis							
Yes	150	0.68(0.35-1.29)	0.23		152	0.65(0.41-1.04)	0.068
None	167	0.38(0.24-0.62)	**4.1e-05**		167	0.44(0.28-0.69)	**0.00021**

Bold values indicate P < 0.05.

### Prognostic significance of CCL14 expression in human cancers

**Figure 2:**
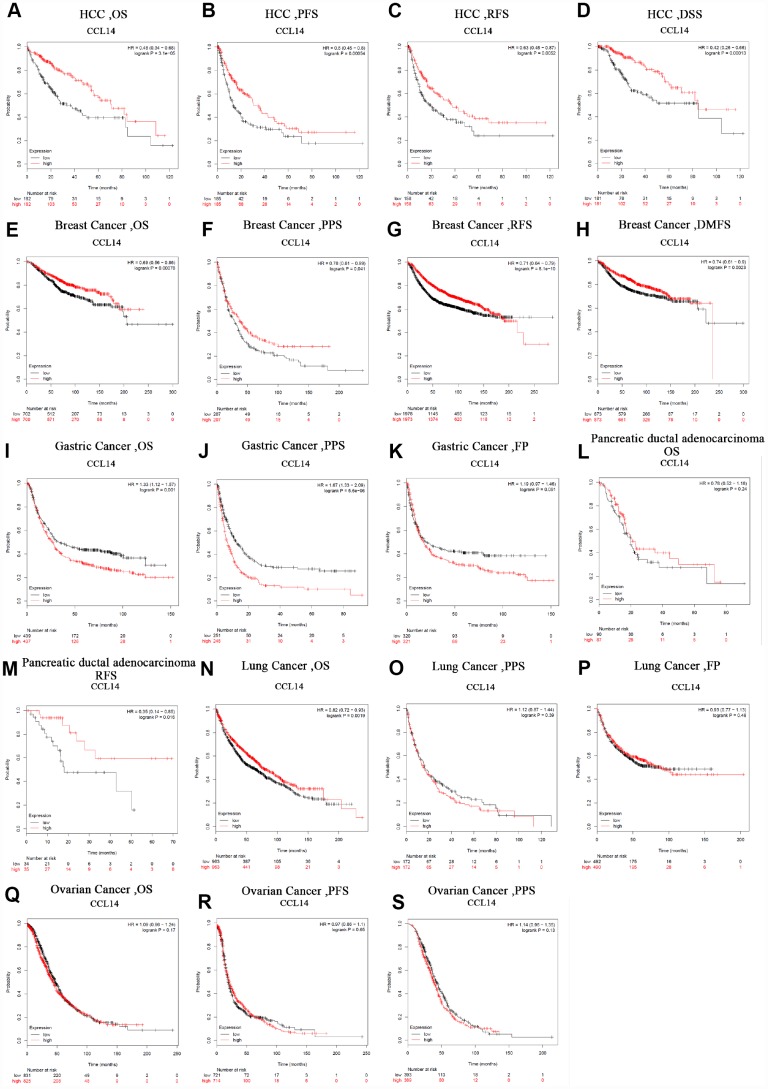
**Kaplan-Meier survival curve analysis of the prognostic significance of high and low expression of CCL14 in different types of human cancers using the Kaplan-Meier plotter database (A-S).** (**A**–**D**) High CCL14 expression was correlated with better OS, PFS, RFS and DSS in HCC cohorts (n=364, n=370, n=316, n=362). (**E**–**H**) Survival curves of OS, PPS, RFS and DMFS in the breast cancer cohort (n=1,404, n=414, n=3,915). (**I**–**K**) OS, PS and DFS survival curves of gastric cancer. High CCL14 expression was correlated with poor OS and PS (n = 876, n = 499, n=641). (**L**, **M**) OS and RFS survival curves of ductal adenocarcinoma (n = 69, n = 177). (**N**–**P**) OS, PPS and FP survival curves of lung cancer (n = 1,926, n = 344, n=982). (**Q**–**S**) OS, PFS and PPS survival curves of ovarian cancer (n = 1,656, n = 1,435, n=782). OS, overall survival; PFS, progression-free survival; RFS, relapse-free survival; DSS, disease-specific survival. DMFS, distant metastasis-free survival; PPS, post progression survival; FP, first progression.

Next, we analyzed the prognostic value of CCL14 expression in human cancers using the Kaplan-Meier plotter database. Low CCL14 expression was associated with poorer prognosis in HCC (OS: HR=0.48, 95% CI=0.34 to 0.68, P=3.1e-05; PFS: HR=0.6, 95% CI=0.45 to 0.8, P =0.00054; RFS: HR=0.63, 95% CI=0.45 to 0.87, P=0.0052; DSS: HR=0.42, 95% CI=0.26 to 0.66, P=0.00013; Figure 2A–2D), breast cancer (OS: HR=0.69, 95% CI=0.56 to 0.86, P=0.00076; PPS: HR=0.78, 95% CI=0.61 to 0.99, P=0.041; RFS: HR =0.71, 95% CI=0.64 to 0.79, P=8.1e-10; DMFS: HR=0.74, 95% CI=0.61 to 0.9, P=0.0023; Figure 2E–2H), gastric cancer (OS: HR=1.33, 95% CI=1.12 to 1.57, P=0.001; PPS: HR=1.67, 95% CI=1.33 to 2.09, P=5.6e-06: Figure 2I–2J), pancreatic ductal adenocarcinoma (RFS: HR=0.35, 95% CI=0.14 to 0.85, P=0.016; Figure 2M), and lung cancer (OS: HR=0.82, 95% CI=0.72 to 0.93, P=0.0019; Figure 2N). However, CCL14 expression was not associated with FP in gastric cancer (Figure 2K), OS in pancreatic ductal adenocarcinoma (Figure 2M), PPS and FP in Lung Cancer (Figure 2O, 2P), and OS, PFS, and PPS in ovarian cancer (Figure 2Q–2S). These results demonstrate the prognostic significance of CCL14 expression in liver, breast, gastric, pancreatic ductal, and lung cancers.

The prognostic potential of the level of CCL14 mRNA expression in various cancers was also analyzed using the TCGA RNA sequencing data in the GEPIA database. Low CCL14 expression levels were associated with poorer OS in LGG and LUAD, poorer DFS in THCA, and poorer OS and DFS in HCC. Moreover, low expression of CCL14 correlated with better OS rates in BLCA, COAD, and STAD (Supplementary Figure 1-1~3). These results demonstrate the prognostic significance of CCL14 expression in several human cancers, though their correlation may vary depending on the cancer type.

### Correlation between CCL14 expression and clinical characteristics of HCC patients

Next, we investigated the relationship between CCL14 expression and different clinical characteristics of HCC using the Kaplan-Meier Plotter database and the results are shown in Table 1. Low CCL14 expression correlated with both poorer OS and PFS in males (OS: HR=0.37, P=1.8e-05; PFS HR=0.56, P=0.0013), Asians (OS: HR=0.3, P=0.00015; PFS HR=0.61, P=0.04), alcohol consumers (OS: HR=0.52, P=0.0048; PFS HR=0.5, P=0.0088) non-alcoholics (OS: HR=0.53, P=0.0068; PFS HR=0.66, P=0.042), and patients without hepatitis viral infection (OS: HR=0.38, P=4.1e-05; PFS HR=0.44, P=0.00021). Specifically, low CCL14 mRNA expression correlates with worse OS and PFS in HCC patients belonging to stages 1+2 (OS: HR=0.52, P=0.0074; PFS: HR=0.64, P=0.019) or grade 2 (OS: HR=0.52, P=0.012; PFS: HR=0.57, P=0.01). However, CCL14 expression did not correlate with OS and PFS in females (OS: HR=0.59, P=0.061; PFS: HR=0.7, P=0.16), grade 1 patients (OS: HR=0.67, P = 0.4; PFS: HR=0.89, P=0.77), patients with vascular invasion (OS: HR=0.46, P=0.05; PFS: HR=0.73, P =0.28), and patients with hepatitis virus (OS: HR=0.68, P=0.23; PFS: HR=0.65, P=0.068). These results demonstrate that prognostic significance of CCL14 expression in HCC patients based on their clinical characteristics, especially in early stage of HCC.

### The levels of CCL14 expression correlate with the infiltration levels of immune cells in hepatocellular carcinoma

**Figure 3:**
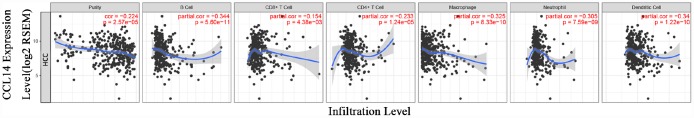
**Correlation analysis of CCL14 expression and infiltration levels of immune cells in HCC tissues using the TIMER database.** CCL14 expression in HCC tissues negatively correlates with tumor purity and infiltration levels of B cells, CD8^+^ T cells, CD4^+^ T cells, macrophages, neutrophils, and dendritic cells (n =371).

The survival times of patients in several cancers is determined by the quantity and activity status of tumor-infiltrating lymphocytes [22, 23]. Therefore, we explored the relationship between CCL14 expression and the infiltrating immune cells in 39 cancer types including HCC using the TIMER database. The results show that CCL14 expression significantly correlates with tumor purity in 32 cancer types. Moreover, CCL14 expression significantly correlates with the infiltration levels of CD4^+^T cells in 28 cancer types, B cells in 19 cancer types, CD8^+^T cells in 15 cancer types, dendritic cells in 16 cancer types, macrophages in 19 cancer types, and neutrophils in 17 cancer types (Figure 3 and Supplementary Figure 2).

Next, we analyzed the association of CCL14 expression with prognosis and immune infiltration in several cancers. The analysis of immune infiltration by genomic methods is highly influenced by tumor purity in clinical samples [24]. Moreover, GEPIA and TIMER databases contain most of the homologous TCGA [25, 26]. Therefore, we selected the cancer types in the TIMER database that show significant correlation between CCL14 expression and tumor purity, and in the GEPIA database that CCL14 expression is relevant to tumor prognosis. We observed that low CCL14 expression correlated with poorer prognosis and high infiltration of most immune cell types in HCC (Figure 3). The level of CCL14 expression negatively correlated with the infiltration levels of B cells (r=-0.344, P=5.60e-11), CD4^+^ T cells (r=-0.233, P=1.24e-05), CD8^+^ T cells (r=-0.154, P=0.004), macrophages (r=-0.325, P=8.33e-10), neutrophils (r=-0.304, P=7.59e-09), and DCs (r=-0.340, P=1.22e-10) in HCC tissues (Figure 3A). However, in LUAD, low CCL14 expression associated with poorer prognosis but positively correlated with infiltration levels of B cells (r=0.250, P=2.57e-08), CD8^+^ T cells (r=0.181, P=6.09e-05), CD4^+^ T cells (r=0.167, P=0.0002), macrophages (r=0.267, P=2.43e-09), neutrophils (r=0.108, P=0.018), and DCs (r=0.172, P=0.0001). In COAD, low CCL14 expression associated with better prognosis and positively correlated with infiltration levels of B cells (r=0.115, P=0.021), CD8^+^ T cells (r=0.184, P=0.0002), CD4^+^ T cells (r=0.293, P=2.14e-09), macrophages (r=0.442, P=1.01e-20), neutrophils (r=0.254, P=2.64e-07), and DCs (r=0.299, P=9.71e-10) as shown in Supplementary Figure 2. Although these findings show variation between the status of tumor infiltration of immune cells, the level of CCL14 expression and prognosis in different cancers, the data suggest that CCL14 expression modulates infiltration of immune cells into tumor tissues.

### Correlation analysis between mRNA levels of CCL14 and markers of different subsets of immune cells

Next, we investigated the correlation between CCL14 expression and the status of tumor-infiltrating immune cells based on the levels of immune marker gene expression in HCC and CHOL tissues using the TIMER and GEPIA databases. The immune cells analyzed in HCC tissues included CD8^+^ T cells, CD4^+^ T cells, B cells, tumor-associated macrophages (TAMs), monocytes, M1 and M2 macrophages, neutrophils, DCs, and natural killer (NK) cells. Moreover, different subsets of T cells, namely, T helper 1 (Th1), Th2, follicular helper T (Tfh), Th17, regulatory T (Tregs), and exhausted T cells were also analyzed. Since tumor purity of clinical samples influences the analysis of immune infiltration, the correlation analysis was adjusted for purity (Table 2).

**Table 2:** Correlation analysis between CCL14 and relate genes and markers of immune cells in TIMER.

**Description**	**Gene markers**	**HCC**						**CHOL**				
		**None**			**Purity**			**None**			**Purity**	
		**Core**	**P**		**Core**	**P**		**Core**	**P**		**Core**	**P**
CD8+ T cell	CD8A	0.053	0.305		-0.037	0.488		0.213	0.212		0.120	0.492
	CD8B	0.047	0.367		-0.04	0.461		0.206	0.227		0.137	0.434
T cell (general)	CD3D	-0.02	0.702		-0.134	*		0.350	*		0.273	0.113
	CD3E	0.111	*		-0.004	0.946		0.427	*		0.364	*
	CD2	0.089	0.087		-0.025	0.640		0.320	0.058		0.229	0.186
B cell	CD19	-0.013	0.808		-0.085	0.115		0.634	***		0.061	0.987
	CD79A	0.106	*		0.006	0.913		0.489	**		0.439	**
Monocyte	CD86	-0.045	0.387		-0.211	***		0.174	0.308		0.06	0.731
	CD115	0.06	0.249		-0.089	0.100		0.209	0.221		0.123	0.480
TAM	CCL2	0.184	***		0.08	0.139		0.255	0.133		0.213	0.219
	CD68	-0.042	0.424		-0.165	**		-0.040	0.815		-0.114	0.513
	IL10	-0.06	0.253		-0.200	***		0.237	0.164		0.109	0.532
M1 Macrophage	iNOS	0.233	***		0.222	***		0.102	0.554		0.106	0.544
	IRF5	0.025	0.637		-0.029	0.594		0.132	0.442		0.074	0.671
	COX2	0.119	*		-0.016	0.763		0.155	0.336		0.071	0.687
M2 Macrophage	CD163	0.149	**		0.045	0.403		0.347	*		0.264	0.126
	VSIG4	0.075	0.147		-0.049	0.360		0.175	0.307		0.081	0.643
	MS4A4A	0.138	**		0.017	0.750		0.289	0.087		0.180	0.301
Neutrophils	CD66b	-0.045	0.383		-0.08	0.136		-0.09	0.602		-0.098	0.576
	CD11b	-0.223	***		-0.347	***		0.148	0.387		0.093	0.594
	CCR7	0.227	***		0.124	*		0.438	**		0.378	*
Natural killer cell	KIR2DL1	0.111	*		0.114	*		0.219	0.199		0.194	0.264
	KIR2DL3	-0.012	0.818		-0.057	0.290		0.391	*		0.373	*
	KIR2DL4	-0.073	0.161		-0.012	0.058		-0.026	0.879		-0.086	0.623
	KIR3DL1	0.095	0.068		0.07	0.197		0.007	0.9652		-0.023	0.896
	KIR3DL2	0.096	0.064		0.053	0.324		0.130	0.451		0.126	0.470
	KIR3DL3	-0.012	0.825		-0.017	0.749		0.105	0.543		0.069	0.694
	KIR2DS4	0.11	*		0.111	*		0.053	0.761		0.012	0.946
Dendritic cell	HLA-DPB1	0.113	*		0.004	0.938		0.313	0.06		0.237	0.171
	HLA-DQB1	0.078	0.133		-0.031	0.567		0.294	0.082		0.235	0.175
	HLA-DRA	0.031	0.551		-0.097	0.071		0.427	**		0.367	*
	HLA-DPA1	0.083	0.109		-0.038	0.477		0.336	*		0.264	0.125
	BDCA-1	0.260	***		0.18	***		0.454	**		0.400	*
	BDCA-4	-0.07	0.179		-0.124	*		0.102	0.551		0.020	0.910
	CD11c	-0.058	0.262		-0.205	***		0.222	0.193		0.115	0.510
Th1	T-bet	0.231	***		0.163	**		0.353	*		0.272	0.114
	STAT4	0.074	0.153		0.007	0.899		0.208	0.222		0.150	0.389
	STAT1	-0.152	**		-0.22	***		-0.03	0.863		-0.088	0.614
	IFN-γ	-0.112	*		-0.187	***		0.123	0.474		0.013	0.942
	TNF-α	-0.091	0.079		-0.232	***		0.321	0.056		0.285	0.097
Th2	GATA3	0.069	0.185		-0.049	0.366		0.311	0.065		0.223	0.199
	STAT6	0.176	***		0.19	***		-0.082	0.634		-0.095	0.587
	STAT5A	0.115	*		0.051	0.347		0.303	0.073		0.272	0.115
	IL13	-0.031	0.553		-0.029	0.589		0.479	**		0.458	0.571
Tfh	BCL6	-0.023	0.663		-0.043	0.430		0.127	0.459		0.099	0.572
	IL21	-0.13	*		-0.173	**		0.255	0.133		0.202	0.244
Th17	STAT3	-0.023	0.652		-0.104	0.054		0.152	0.375		0.133	0.447
	IL17A	-0.026	0.614		-0.045	0.403		0.05	0.774		-0.011	0.950
Treg	FOXP3	0.067	0.196		0.041	0.448		0.255	0.133		0.156	0.372
	CCR8	0.186	***		0.181	***		0.320	0.057		0.247	0.153
	STAT5B	0.108	*		0.152	**		0.321	0.057		0.290	0.091
	TGFβ	-0.052	0.314		-0.173	**		0.216	0.204		0.158	0.363
T cell exhaustion	PD-1	-0.093	0.072		-0.191	***		0.209	0.220		0.153	0.379
	CTLA4	-0.134	**		-0.257	***		0.142	0.406		0.067	0.701
	LAG3	0.000	0.995		-0.04	0.458		0.193	0.259		0.123	0.481
	TIM-3	-0.107	*		-0.285	***		0.143	0.403		0.038	0.827
	GZMB	0.183	***		0.123	*		0.194	0.256		0.101	0.564

HCC: hepatocellular carcinoma; CHOL: cholangiocarcinoma; TAM: tumor-associated macrophage; Th: T helper cell; Tfh: Follicular helper T cell; Treg, regulatory T cell; Cor, R value of Spearman’s correlation; None, correlation without adjustment. Purity, correlation adjusted by purity.*P<0.05, **P<0.01, ***P<0.001

**Figure 4:**
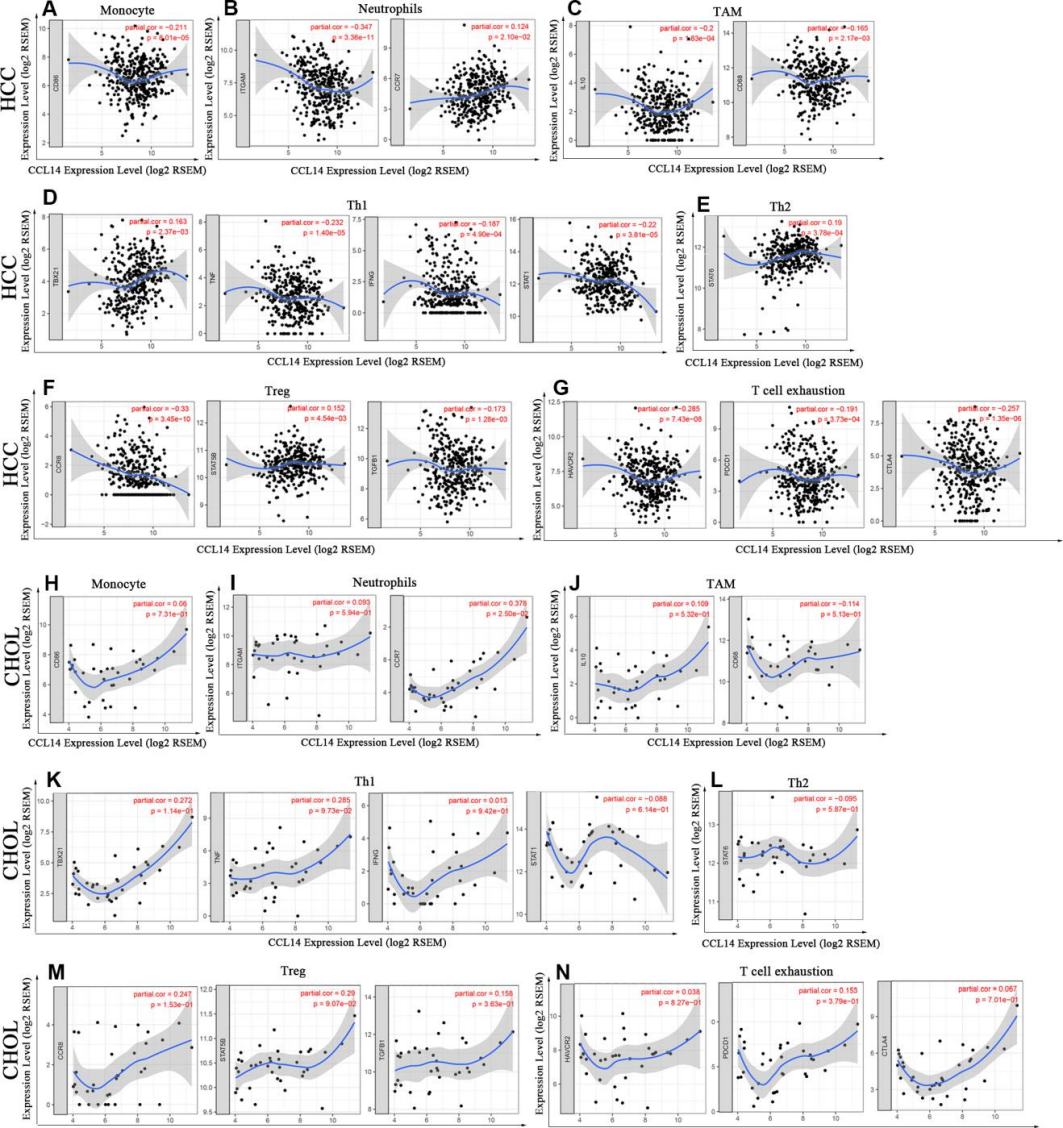
**Correlation analysis of CCL14 expression and the expression of marker genes of infiltrating immune cells in HCC (A-G) and CHOL (H-N) using the TIMER database.** (**A**–**G**) The scatter plots show correlation between CCL14 expression and the gene markers of (**A**) Monocytes (CD86); (**B**) Neutrophils (ITGAM and CCR7); (**C**) TAMs (IL-10 and CD68); (**D**) Th1 cells (TBX21, TNF, IFNG and STAT1); (**E**) Th2 cells (STAT6); (**F**) Tregs (CCR8, STAT5B and TGFB1); and (**G**) Exhausted T cells (HAVCR2, PDCD1 and CTLA4) in HCC samples (n = 371). (**H**–**N**) The scatter plots show correlations between CCL14 expression and the gene markers of (**H**) Monocytes (CD86); (**I**) Neutrophils (ITGAM and CCR7); (**J**) TAMs (IL-10 and CD68); (**K**)Th1 cells (TBX21, TNF, IFNG and STAT1); (**L**) Th2 cells (STAT6); (**M**) Tregs (CCR8, STAT5B and TGFB1); and (**N**) Exhausted T cells (HAVCR2, PDCD1 and CTLA4) in CHOL (n = 36).

Analysis of the TIMER and GEPIA databases showed that CCL14 expression in HCC tissues significantly correlated with the expression of marker genes from tumor-infiltrating monocytes, TAMs, neutrophils, DCs, T-helper, Treg and exhausted T cells, whereas the correlation was not significant in CHOL (Figure 4 and Table 3).

**Table 3:** Correlation analysis between CCL14 and marker genes of immune cells in GEPIA.

**Description**	**Gene markers**	**HCC**						**CHOL**				
		**Tumor**			**Normal**			**Tumor**			**Normal**	
		**R**	**P**		**R**	**P**		**R**	**P**		**R**	**P**
Monocyte	CD86	−0.083	0.11		−0.0029	0.98		0.19	0.28		0.67	0.059
Neutrophils	CD11b	−0.2	**0.00012**		0.0031	0.98		−0.06	0.73		0.68	0.05
	CCR7	0.19	**0.00017**		−0.0029	0.98		0.42	**0.011**		0.81	**0.0079**
TAM	CD68	−0.15	**0.0043**		0.26	0.069		−0.049	0.78		0.87	**0.0045**
	IL-10	−0.021	0.68		0.05	0.73		0.13	0.45		0.042	0.91
Th1	IFN-γ (IFNG)	−0.18	**0.00062**		0.22	0.12		−0.00058	1		0.12	0.76
	STAT1	−0.16	**0.0018**		−0.042	0.77		−0.069	0.69		0.13	0.74
	T-bet (TBX21)	0.14	**0.0053**		0.13	0.36		0.3	0.074		0.67	0.059
	TNF-α (TNF)	−0.091	**0.081**		−0.019	0.9		0.29	0.086		0.34	0.38
Th2	STAT6	−0.0051	0.92		0.069	0.63		−0.15	0.39		0.77	**0.021**
Treg	CCR8	−0.13	**0.011**		−0.072	0.62		0.33	**0.047**		0.26	0.5
	STAT5B	−0.075	0.15		0.17	0.23		0.14	0.41		0.73	**0.031**
	TGF-β (TGFB1)	−0.065	0.21		0.2	0.17		0.18	0.3		0.82	**0.011**
T cell exhaustion	CTLA4	−0.21	**0.000043**		−0.12	0.43		0.13	0.44		0.67	**0.049**
	PD-1 (PDCD1)	−0.12	**0.021**		−0.038	0.79		0.19	0.26		0.81	**0.0086**
	TIM-3 (HAVCR2)	−0.13	**0.013**		0.37	**0.0087**		0.044	0.8		0.93	**0.00075**

Specifically, CCL14 expression showed significant correlation with the expression of markers of specific immune cells such as monocyte marker, CD86 (r=-0.211; P=8.01e-05), TAM markers, CD68 (r=-0.165; P=0.002), and IL-10 (r=-0.2; P=1.83e-04), M1 macrophage marker, iNOS (r=0.222; P=3.27e-05), neutrophil markers, CD11b (r=-0.347; P=3.36e-11), and CCR7 (r= 0.124; P=2.10e-02), NK cell markers, KIR2DL1 (r=0.114; P=3.46e-02), and KIR2DS4 (r=0.111; P=3.86e-02), and DC markers, BDCA-1 (r=0.18; P=8.00e-04), BDCA-4 (r= -0.124; P=2.16e-02), and CD11c (r= 0.205; P= 1.22e-04).

The expression of CCL14 correlated significantly with the expression of the marker genes of different subsets of T cells in HCC, namely, Th1 markers, T-bet (r=0.163; P=2.37e-03), STAT-1 (r=-0.22; P=3.81e-05), IFN-γ (r=-0.187; P=4.90e-04), and TNF-α (r=0.232, P=1.40e-05), Th2 marker, STAT-6 (r=0.19, p=3.78e-04), Tfh marker, IL-21 (r=-0.173, p=1.28e-03), and Treg markers, CCR8 (r=0.181, p=3.45e-10), STAT5B (r=0.152, p=4.54e-03), and TGF-β (r=-0.173, p=1.28e-03). Furthermore, we observed significant negative correlation between the expression of CCL14 and the expression of exhausted T cell markers in HCC, namely, PD-1 (r=-0.191; p=3.73e-04), CTLA-4 (r=-0.257; p=1.35e-06), and TIM-3 (r=-0.285; p= 7.43e-08). CCL14 expression did not show any significant correlation with the expression of marker genes for CD8^+^ and general T cells, B cells, and Th17 cells in HCC. These findings strongly suggest that CCL14 expression correlates with infiltration of immune cells in hepatocellular carcinoma.

## DISCUSSION

This study demonstrates that CCL14 mRNA levels correlate with prognosis of several human cancers. Low CCL14 mRNA levels correlate with a worse prognosis in several cancers, including HCC, breast cancer, lung cancer, and pancreatic ductal adenocarcinoma. The downregulation of CCL14 associated with worse prognosis in patients associated with clinical characteristics such as males, Asians, alcohol consumers, those in early stages of tumor (stage 1+2), and those without hepatitis viral infections. Furthermore, CCL14 mRNA levels correlate with the numbers of tumor-infiltrated immune cells based on the levels of markers for different immune cell types in HCC. Our study suggests that CCL14 is a potential prognostic biomarker for HCC and other cancers.

The levels of CCL14 mRNA in cancer tissues were analyzed using the ONCOMINE, TIMER, Kaplan-Meier Plotter, and GEPIA databases. Analysis of CCL14 mRNA levels in cancer and normal tissues in the ONCOMINE and the TIMER databases revealed that CCL14 expression was significantly downregulated in most cancers. However, there was variability in the expression of CCL14 in different types of cancers, which may reflect differences in the data collection methods and the underlying causative mechanisms. However, the CCL14 expression data was consistent in HCC tissues across different databases. Gene expression analysis of the GEPIA database revealed that low CCL14 expression correlated with worse prognosis in the cancer types such as HCC, CHOL, LGG, LUAD, and THCA. Furthermore, Kaplan-Meier Plotter analyses showed that low CCL14 expression correlated with worse prognosis in liver, lung, breast and pancreatic ductal cancers. In HCC patients, low CCL14 expression correlated with worse prognosis of patients in early stages (stage 1+2 or grade 2), and better OS and PFS in HCC patients with high CCL14 expression (Table 1). These data strongly suggest that CCL14 is a potential prognostic biomarker in HCC, especially for patients in early cancer stages.

This study also demonstrates that CCL14 expression correlated with the infiltration status of immune cells in several cancer types, including HCC. In HCC, there was a strong negative correlation between CCL14 expression with infiltration of B cells, CD4^+^ and CD8^+^ T cells, macrophages, neutrophils, and DCs. This suggests that CCL14 plays an important role in regulating tumor immunity, and therefore influences HCC prognosis. We observed correlation between the levels of CCL14 mRNA and the expression of the monocyte marker, CD86, TAM markers, CD68 and IL-10, and M1 macrophage marker, iNOS. This suggests that CCL14 regulates infiltration and activity of tumor-associated macrophages (TAM). CCL14 expression also correlates with the expression of markers of different subsets of T helper (Th) cells, including Th1 (T-bet, STAT-1, IFN-γ and TNF-α), Th2 (STAT6), Tfh (IL-21), and Tregs (CCR8, STAT5B and TGF-β). This suggests a role for CCL14 in regulating tumor-infiltration of T-helper cells. Moreover, expression of exhausted T cells markers, PD-1, CTLA-4 and TIM-3, which are critical inhibitory immune checkpoint proteins negatively correlate with CCL14 expression. Most cancers, including HCC, overexpress inhibitory ligands to evade immune response by dampening T cell function, thus contributing to cancer progression [27, 28]. The expression of inhibitory immune checkpoint proteins is altered in the tumor microenvironment [29]. CCL14 can bind to chemokine receptors, such as CCR1, CCR3, and CCR5, and regulate activation and migration of different leukocytes by mobilizing Ca^2+^ influx [30, 31]. Altered Ca^2+^ flux in the T cell subsets promotes cytokine production and downregulates CTLA-4 and PD-1 expression [32]. We postulate that low CCL14 expression in the tumor microenvironment diminishes the Ca^2+^ influx and upregulates the expression of inhibitory immune checkpoint proteins, PD-1, CTLA-4 and TIM-3 on the exhausted T cells. These mechanistic changes can alter anti-tumor function of T cells and result in poorer prognosis of HCC. However, this hypothesis needs to be further investigated. Taken together, our findings indicate that CCL14 plays an important role in regulating tumor-infiltration of immune cells in HCC.

Immune responses at primary and secondary tumor sites depend on the different types of immune cells that infiltrate into the tumor micro­environment. The infiltration of different types of immune cells is tightly regulated by the various chemokines, which modulate tumor immunity and the biological phenotype of the tumors, and also influence tumor progression, therapy and prognosis [33–35]. Most members of the CC family play important roles in initiation, growth, and metastasis of cancers [36, 37]. For example, high expression of CXCL9 and CXCL10 is associated with increased numbers of tumor infiltrating CD8^+^ T cells, decreased metastasis, and improved survival in patients with ovarian and colon cancers [38]. High expression of CC­chemokine receptor 6 (CCR6), CXCR4, multiple CD49 integrins, and the C­type lectin­like receptor, CD161, are associated with Th17 cell migration and retention within inflamed tissues and tumors [39]. CXCL8 is a key mediator that recruits Treg cells into the tumor microenvironment [17].

Although CCL14 belongs to the CC family of chemokines, little is known about its role in HCC progression. CCL14 promotes activation of monocytes, eosinophils, and T lymphoblasts during HIV infection [30]. It is a critical mediator of the JARID1B/ LSD1/NuRD complex, which regulates angiogenesis and metastasis in breast cancer patients, and increases the proliferation and migration of lung cancer cells [40]. Moreover, CCL14 promotes growth and survival of macrophages by activating the PI3K/Akt and ERK signaling pathways and increasing c-myc expression [41]. Our study shows that low CCL14 expression is associated with poorer prognosis in HCC, and infiltration of various types of immune cells, including B cells, DCs, macrophages, neutrophils, CD4^+^, and CD8^+^ T cells. CCL14 expression also correlates with infiltration of Th, Treg, and exhausted T cells. Hence, our study suggests that CCL14 is a potential independent biomarker for HCC prognosis and the status of tumor immunity.

Several factors could influence the outcomes of this study. Firstly, this study is based on data retrieved from published articles, public repositories, and communications with study authors. Hence, the quality of data can influence the study outcomes. Secondly, the quantity of samples in the databases is constantly supervised and extended, which can affect the outcomes of this study. Thirdly, the accuracy and choice of the statistical methods used by the databases to analyze the data could affect the interpretation of the study results. However, we obtained similar results by analyzing multiple databases, which supports the conclusions of our study.

Our study has some limitations. Firstly, our investigations into the role of CCL14 in tumors were based on data that was already reported in the ONCOMINE, Kaplan-Meier plotter, GEPIA and TIMER databases. However, we did not verify these outcomes by testing our own clinical samples. Secondly, the sample sizes of some individual tumors in the databases were small. In such cases, large sample sizes will be necessary for reliable interpretation of data. Thirdly, we did not conduct *in vitro* and animal experiments to confirm the role of CCL14 in the growth and progression of HCC, and its relationship with the infiltration of immune cells into the tumor microenvironment. Hence, further studies are necessary to verify the role played by CCL14 in HCC.

In summary, our results suggest that CCL14 is a potential independent prognostic biomarker for HCC that can be used to evaluate the levels of immune cell infiltration in the tumor tissues. Relatively low levels of CCL14 in HCC and other cancer tissues may indicate greater risk of tumor relapse after treatment and close medical supervision will be necessary for such patients.

## MATERIALS AND METHODS

### CCL14 gene expression analysis

The mRNA levels of CCL14 in several cancers including HCC were identified from the Oncomine database (https://www.oncomine.org/resource/login.html) [42]. The threshold was determined as follows: fold change of 1.5, P-value of 0.001, and gene ranking of all.

### Kaplan-Meier survival curve analysis

Kaplan-Meier survival curve analysis was performed to assess the correlation between the expression of the 54,000 genes on the survival rates in 21 different cancers using more than 10,000 cancer samples, including 371 liver, 1440 gastric, 3452 lung, 2190 ovarian, and 6234 breast cancer samples. Kaplan-Meier plots (http://kmplot.com/analysis/) were used to analyze the relationship between CCL14 gene expression and survival rates in liver, gastric, breast, pancreatic, ovarian, and lung cancers based on the hazard ratios (HR) and log-rank P-values [43].

### TIMER analysis

TIMER database was used to systematically analyze the tumor-infiltrating immune cells (TIICs) in 32 cancer types using more than 10,000 samples from The Cancer Genome Atlas (TCGA) (https://cistrome.shinyapps.io/timer/) database [25]. TIMER determines the abundance of tumor-infiltrating immune cells (TIICs) based on the statistical analysis of gene expression profiles [44]. We analyzed the association between the level of CCL14 gene expression and the abundance of infiltrating immune cells, including CD4^+^ T cells, CD8^+^ T cells, B cells, neutrophils, dendritic cells and macrophages based on expression of specific marker genes in different cancers including HCC. The marker genes used for analysis of tumor-infiltrating immune cells including T cells, B cells, TAMs, monocytes, M1 macrophages, M2 macrophages, natural killer (NK) cells, neutrophils, dendritic cells (DCs), T-helper (Th) cells, T-helper 17 (Th17) cells, follicular helper T (Tfh) cells, exhausted T cells, and Tregs were based on data from previous studies [45, 46]. CCL14 gene was on the x-axis and related marker genes are on the y-axis.

### GEPIA analysis

The Gene Expression Profiling Interactive Analysis (GEPIA) database (http://gepia.cancer-pku.cn/index.html) was used to analyze the RNA sequencing expression data from 8,587 normal and 9,736 tumor tissue samples from the TCGA and GTEx projects [26]. We also used GEPIA to generate survival curves and determine OS and DFS rates and their correlation to specific gene expression in 33 different types of cancer to further confirm the significantly correlated genes in the TIMER analysis.

### Statistical analysis

Gene expression data from the Oncomine database were analyzed using the P-values, fold changes, and ranks. Survival curves were produced by the Kaplan-Meier plots and GEPIA database. The correlation of gene expression was evaluated in the TIMER and GEPIA databases using Spearman’s correlation analysis. P-values <0.05 were considered as statistically significant.

## Supplementary Material

Supplementary Figures

Supplementary Table 1

## References

[1] Heimbach JK, Kulik LM, Finn RS, Sirlin CB, Abecassis MM, Roberts LR, Zhu AX, Murad MH, Marrero JA. AASLD guidelines for the treatment of hepatocellular carcinoma. Hepatology. 2018; 67:358–80. 10.1002/hep.29086 28130846

[2] Makarova-Rusher OV, Altekruse SF, McNeel TS, Ulahannan S, Duffy AG, Graubard BI, Greten TF, McGlynn KA. Population attributable fractions of risk factors for hepatocellular carcinoma in the United States. Cancer. 2016; 122:1757–65. 10.1002/cncr.29971 26998818 PMC5548177

[3] Zhang BH, Yang BH, Tang ZY. Randomized controlled trial of screening for hepatocellular carcinoma. J Cancer Res Clin Oncol. 2004; 130:417–22. 10.1007/s00432-004-0552-0 15042359 PMC12161851

[4] Huang J, Yan L, Cheng Z, Wu H, Du L, Wang J, Xu Y, Zeng Y. A randomized trial comparing radiofrequency ablation and surgical resection for HCC conforming to the Milan criteria. Ann Surg. 2010; 252:903–12. 10.1097/SLA.0b013e3181efc656 21107100

[5] Feng K, Yan J, Li X, Xia F, Ma K, Wang S, Bie P, Dong J. A randomized controlled trial of radiofrequency ablation and surgical resection in the treatment of small hepatocellular carcinoma. J Hepatol. 2012; 57:794–802. 10.1016/j.jhep.2012.05.007 22634125

[6] Llovet JM, Ricci S, Mazzaferro V, Hilgard P, Gane E, Blanc JF, de Oliveira AC, Santoro A, Raoul JL, Forner A, Schwartz M, Porta C, Zeuzem S, et al, and SHARP Investigators Study Group. Sorafenib in advanced hepatocellular carcinoma. N Engl J Med. 2008; 359:378–90. 10.1056/NEJMoa0708857 18650514

[7] Bruix J, Raoul JL, Sherman M, Mazzaferro V, Bolondi L, Craxi A, Galle PR, Santoro A, Beaugrand M, Sangiovanni A, Porta C, Gerken G, Marrero JA, et al. Efficacy and safety of sorafenib in patients with advanced hepatocellular carcinoma: subanalyses of a phase III trial. J Hepatol. 2012; 57:821–29. 10.1016/j.jhep.2012.06.014 22727733 PMC12261288

[8] El-Serag HB, Rudolph KL. Hepatocellular carcinoma: epidemiology and molecular carcinogenesis. Gastroenterology. 2007; 132:2557–76. 10.1053/j.gastro.2007.04.061 17570226

[9] Mazzanti R, Gramantieri L, Bolondi L. Hepatocellular carcinoma: epidemiology and clinical aspects. Mol Aspects Med. 2008; 29:130–43. 10.1016/j.mam.2007.09.008 18061252

[10] Giannelli G, Rani B, Dituri F, Cao Y, Palasciano G. Moving towards personalised therapy in patients with hepatocellular carcinoma: the role of the microenvironment. Gut. 2014; 63:1668–76. 10.1136/gutjnl-2014-307323 25053718

[11] Marra F, Tacke F. Roles for chemokines in liver disease. Gastroenterology. 2014; 147:577–594.e1. 10.1053/j.gastro.2014.06.043 25066692

[12] Chiu DK, Xu IM, Lai RK, Tse AP, Wei LL, Koh HY, Li LL, Lee D, Lo RC, Wong CM, Ng IO, Wong CC. Hypoxia induces myeloid-derived suppressor cell recruitment to hepatocellular carcinoma through chemokine (C-C motif) ligand 26. Hepatology. 2016; 64:797–813. 10.1002/hep.28655 27228567

[13] Nagarsheth N, Wicha MS, Zou W. Chemokines in the cancer microenvironment and their relevance in cancer immunotherapy. Nat Rev Immunol. 2017; 17:559–72. 10.1038/nri.2017.49 28555670 PMC5731833

[14] Griffith JW, Sokol CL, Luster AD. Chemokines and chemokine receptors: positioning cells for host defense and immunity. Annu Rev Immunol. 2014; 32:659–702. 10.1146/annurev-immunol-032713-120145 24655300

[15] Pagès F, Berger A, Camus M, Sanchez-Cabo F, Costes A, Molidor R, Mlecnik B, Kirilovsky A, Nilsson M, Damotte D, Meatchi T, Bruneval P, Cugnenc PH, et al. Effector memory T cells, early metastasis, and survival in colorectal cancer. N Engl J Med. 2005; 353:2654–66. 10.1056/NEJMoa051424 16371631

[16] Galon J, Costes A, Sanchez-Cabo F, Kirilovsky A, Mlecnik B, Lagorce-Pagès C, Tosolini M, Camus M, Berger A, Wind P, Zinzindohoué F, Bruneval P, Cugnenc PH, et al. Type, density, and location of immune cells within human colorectal tumors predict clinical outcome. Science. 2006; 313:1960–64. 10.1126/science.1129139 17008531

[17] Kryczek I, Wang L, Wu K, Li W, Zhao E, Cui T, Wei S, Liu Y, Wang Y, Vatan L, Szeliga W, Greenson JK, Roliński J, et al. Inflammatory regulatory T cells in the microenvironments of ulcerative colitis and colon carcinoma. Oncoimmunology. 2016; 5:e1105430. 10.1080/2162402X.2015.1105430 27622054 PMC5007964

[18] Kryczek I, Wu K, Zhao E, Wei S, Vatan L, Szeliga W, Huang E, Greenson J, Chang A, Roliński J, Radwan P, Fang J, Wang G, Zou W. IL-17+ regulatory T cells in the microenvironments of chronic inflammation and cancer. J Immunol. 2011; 186:4388–95. 10.4049/jimmunol.1003251 21357259

[19] Schulz-Knappe P, Mägert HJ, Dewald B, Meyer M, Cetin Y, Kubbies M, Tomeczkowski J, Kirchhoff K, Raida M, Adermann K, Kist A, Reinecke M, Sillard R, et al. HCC-1, a novel chemokine from human plasma. J Exp Med. 1996; 183:295–99. 10.1084/jem.183.1.295 8551235 PMC2192403

[20] Zlotnik A, Yoshie O. The chemokine superfamily revisited. Immunity. 2012; 36:705–16. 10.1016/j.immuni.2012.05.008 22633458 PMC3396424

[21] Zhang X, Wan JX, Ke ZP, Wang F, Chai HX, Liu JQ. TMEM88, CCL14 and CLEC3B as prognostic biomarkers for prognosis and palindromia of human hepatocellular carcinoma. Tumour Biol. 2017; 39:1010428317708900. 10.1177/1010428317708900 28718365

[22] Japanese Gastric Cancer Association. Japanese gastric cancer treatment guidelines 2014 (ver. 4). Gastric Cancer. 2017; 20:1–19. 10.1007/s10120-016-0622-4 27342689 PMC5215069

[23] Ohtani H. Focus on TILs: prognostic significance of tumor infiltrating lymphocytes in human colorectal cancer. Cancer Immun. 2007; 7:4. 17311363 PMC2935759

[24] Yoshihara K, Shahmoradgoli M, Martínez E, Vegesna R, Kim H, Torres-Garcia W, Treviño V, Shen H, Laird PW, Levine DA, Carter SL, Getz G, Stemke-Hale K, et al. Inferring tumour purity and stromal and immune cell admixture from expression data. Nat Commun. 2013; 4:2612. 10.1038/ncomms3612 24113773 PMC3826632

[25] Li T, Fan J, Wang B, Traugh N, Chen Q, Liu JS, Li B, Liu XS. TIMER: A Web Server for Comprehensive Analysis of Tumor-Infiltrating Immune Cells. Cancer Res. 2017; 77:e108–10. 10.1158/0008-5472.CAN-17-0307 29092952 PMC6042652

[26] Tang Z, Li C, Kang B, Gao G, Li C, Zhang Z. GEPIA: a web server for cancer and normal gene expression profiling and interactive analyses. Nucleic Acids Res. 2017; 45:W98–102. 10.1093/nar/gkx247 28407145 PMC5570223

[27] Granier C, De Guillebon E, Blanc C, Roussel H, Badoual C, Colin E, Saldmann A, Gey A, Oudard S, Tartour E. Mechanisms of action and rationale for the use of checkpoint inhibitors in cancer. ESMO Open. 2017; 2:e000213. 10.1136/esmoopen-2017-000213 28761757 PMC5518304

[28] Rotte A, Jin JY, Lemaire V. Mechanistic overview of immune checkpoints to support the rational design of their combinations in cancer immunotherapy. Ann Oncol. 2018; 29:71–83. 10.1093/annonc/mdx686 29069302

[29] Philips GK, Atkins M. Therapeutic uses of anti-PD-1 and anti-PD-L1 antibodies. Int Immunol. 2015; 27:39–46. 10.1093/intimm/dxu095 25323844

[30] Detheux M, Ständker L, Vakili J, Münch J, Forssmann U, Adermann K, Pöhlmann S, Vassart G, Kirchhoff F, Parmentier M, Forssmann WG. Natural proteolytic processing of hemofiltrate CC chemokine 1 generates a potent CC chemokine receptor (CCR)1 and CCR5 agonist with anti-HIV properties. J Exp Med. 2000; 192:1501–08. 10.1084/jem.192.10.1501 11085751 PMC2193185

[31] Blain KY, Kwiatkowski W, Zhao Q, La Fleur D, Naik C, Chun TW, Tsareva T, Kanakaraj P, Laird MW, Shah R, George L, Sanyal I, Moore PA, et al. Structural and functional characterization of CC chemokine CCL14. Biochemistry. 2007; 46:10008–15. 10.1021/bi700936w 17691823

[32] Rodríguez-Perea AL, Rojas M, Velilla-Hernández PA. High concentrations of atorvastatin reduce in-vitro function of conventional T and regulatory T cells. Clin Exp Immunol. 2019; 196:237–48. 10.1111/cei.13260 30638266 PMC6468172

[33] Balkwill F. Cancer and the chemokine network. Nat Rev Cancer. 2004; 4:540–50. 10.1038/nrc1388 15229479

[34] Zou W. Immunosuppressive networks in the tumour environment and their therapeutic relevance. Nat Rev Cancer. 2005; 5:263–74. 10.1038/nrc1586 15776005

[35] Crespo J, Sun H, Welling TH, Tian Z, Zou W. T cell anergy, exhaustion, senescence, and stemness in the tumor microenvironment. Curr Opin Immunol. 2013; 25:214–21. 10.1016/j.coi.2012.12.003 23298609 PMC3636159

[36] Vilgelm AE, Richmond A. Chemokines Modulate Immune Surveillance in Tumorigenesis, Metastasis, and Response to Immunotherapy. Front Immunol. 2019; 10:333. 10.3389/fimmu.2019.00333 30873179 PMC6400988

[37] Mollica Poeta V, Massara M, Capucetti A, Bonecchi R. Chemokines and Chemokine Receptors: New Targets for Cancer Immunotherapy. Front Immunol. 2019; 10:379. 10.3389/fimmu.2019.00379 30894861 PMC6414456

[38] Zhao E, Maj T, Kryczek I, Li W, Wu K, Zhao L, Wei S, Crespo J, Wan S, Vatan L, Szeliga W, Shao I, Wang Y, et al. Cancer mediates effector T cell dysfunction by targeting microRNAs and EZH2 via glycolysis restriction. Nat Immunol. 2016; 17:95–103. 10.1038/ni.3313 26523864 PMC4684796

[39] Kryczek I, Banerjee M, Cheng P, Vatan L, Szeliga W, Wei S, Huang E, Finlayson E, Simeone D, Welling TH, Chang A, Coukos G, Liu R, Zou W. Phenotype, distribution, generation, and functional and clinical relevance of Th17 cells in the human tumor environments. Blood. 2009; 114:1141–49. 10.1182/blood-2009-03-208249 19470694 PMC2723011

[40] Li Q, Shi L, Gui B, Yu W, Wang J, Zhang D, Han X, Yao Z, Shang Y. Binding of the JmjC demethylase JARID1B to LSD1/NuRD suppresses angiogenesis and metastasis in breast cancer cells by repressing chemokine CCL14. Cancer Res. 2011; 71:6899–908. 10.1158/0008-5472.CAN-11-1523 21937684

[41] Li Y, Zheng Y, Li T, Wang Q, Qian J, Lu Y, Zhang M, Bi E, Yang M, Reu F, Yi Q, Cai Z. Chemokines CCL2, 3, 14 stimulate macrophage bone marrow homing, proliferation, and polarization in multiple myeloma. Oncotarget. 2015; 6:24218–29. 10.18632/oncotarget.4523 26155942 PMC4695181

[42] Rhodes DR, Kalyana-Sundaram S, Mahavisno V, Varambally R, Yu J, Briggs BB, Barrette TR, Anstet MJ, Kincead-Beal C, Kulkarni P, Varambally S, Ghosh D, Chinnaiyan AM. Oncomine 3.0: genes, pathways, and networks in a collection of 18,000 cancer gene expression profiles. Neoplasia. 2007; 9:166–80. 10.1593/neo.07112 17356713 PMC1813932

[43] Lánczky A, Nagy Á, Bottai G, Munkácsy G, Szabó A, Santarpia L, Győrffy B. miRpower: a web-tool to validate survival-associated miRNAs utilizing expression data from 2178 breast cancer patients. Breast Cancer Res Treat. 2016; 160:439–46. 10.1007/s10549-016-4013-7 27744485

[44] Li B, Severson E, Pignon JC, Zhao H, Li T, Novak J, Jiang P, Shen H, Aster JC, Rodig S, Signoretti S, Liu JS, Liu XS. Comprehensive analyses of tumor immunity: implications for cancer immunotherapy. Genome Biol. 2016; 17:174. 10.1186/s13059-016-1028-7 27549193 PMC4993001

[45] Danaher P, Warren S, Dennis L, D’Amico L, White A, Disis ML, Geller MA, Odunsi K, Beechem J, Fling SP. Gene expression markers of Tumor Infiltrating Leukocytes. J Immunother Cancer. 2017; 5:18. 10.1186/s40425-017-0215-8 28239471 PMC5319024

[46] Sousa S, Määttä J. The role of tumour-associated macrophages in bone metastasis. J Bone Oncol. 2016; 5:135–38. 10.1016/j.jbo.2016.03.004 27761375 PMC5063225

