# Supplementary material for: CCL14 is a prognostic biomarker and correlates with immune infiltrates in hepatocellular carcinoma
Source: Aging (Albany NY). 2020 Jan 12;12(1):784–807. doi: 10.18632/aging.102656 (PMC6977663; doi:10.18632/aging.102656)
Supplement: Supplementary Figures [file aging-12-102656-s002..pdf]

SUPPLEMENTARY FIGURES

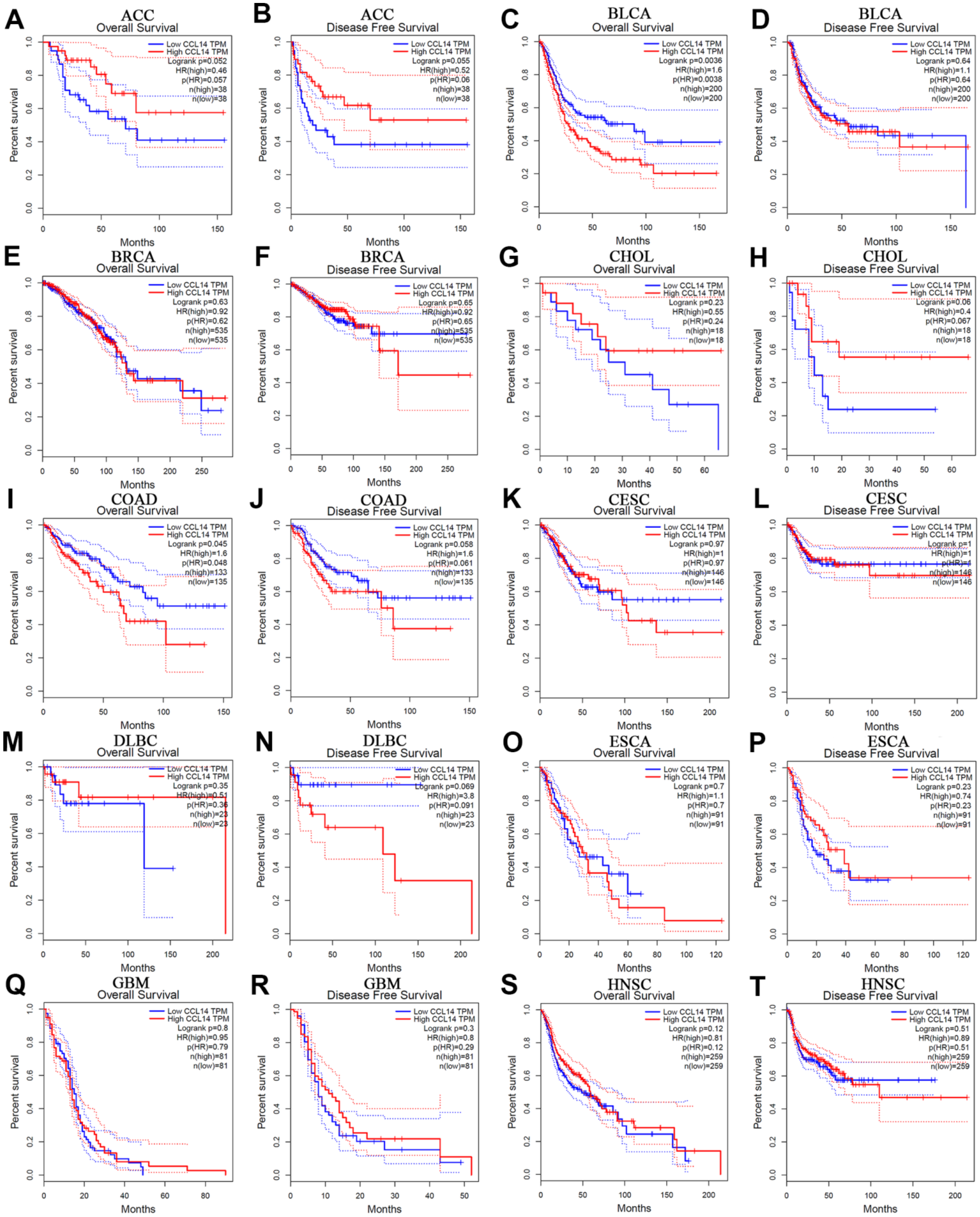

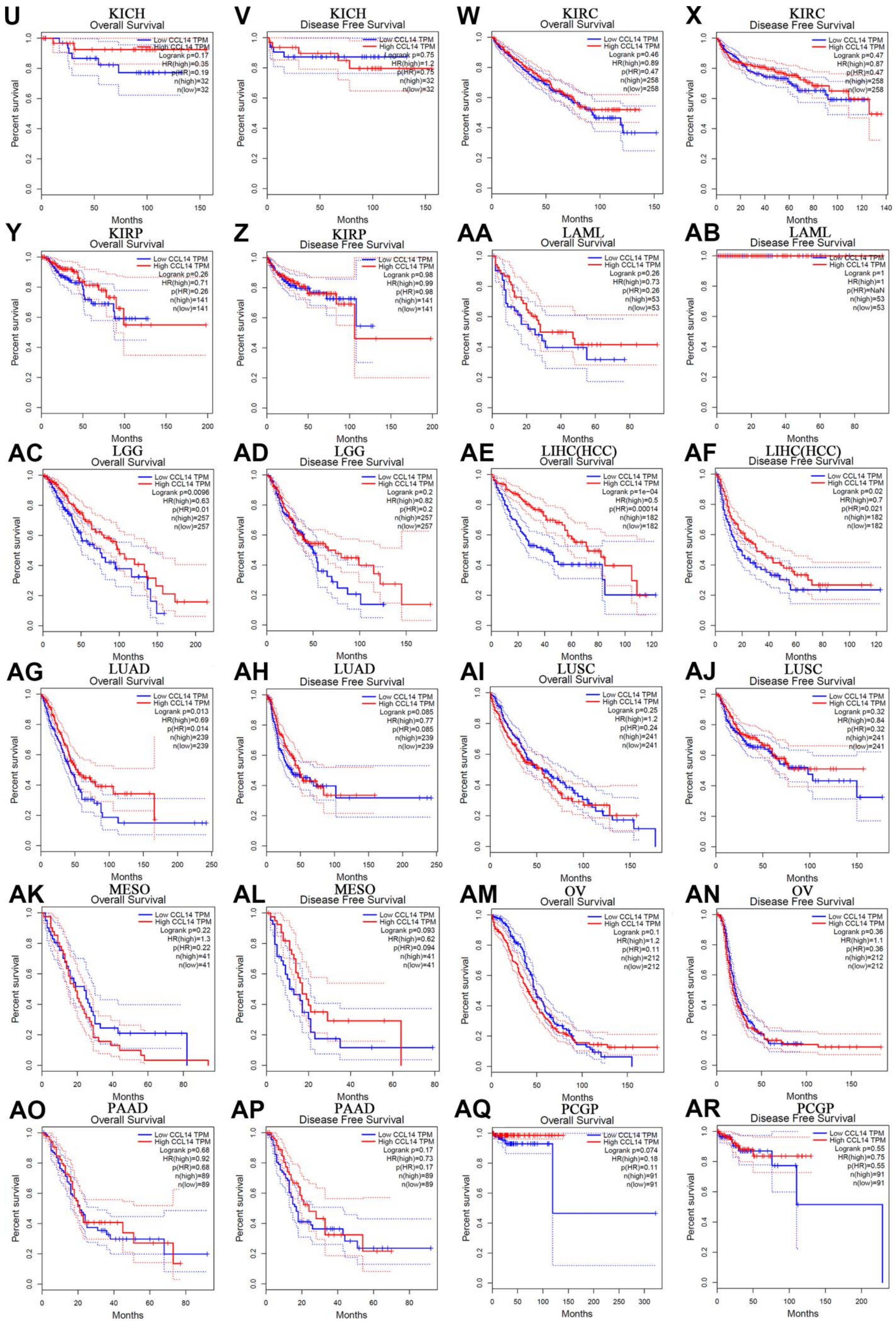

U KICH Overall Survival
V KICH Disease Free Survival
W KIRC Overall Survival
X KIRC Disease Free Survival
Y KIRP Overall Survival
Z KIRP Disease Free Survival
AA LAML Overall Survival
AB LAML Disease Free Survival
AC LGG Overall Survival
AD LGG Disease Free Survival
AE LIHC(HCC) Overall Survival
AF LIHC(HCC) Disease Free Survival
AG LUAD Overall Survival
AH LUAD Disease Free Survival
AI LUSC Overall Survival
AJ LUSC Disease Free Survival
AK MESO Overall Survival
AL MESO Disease Free Survival
AM OV Overall Survival
AN OV Disease Free Survival
AO PAAD Overall Survival
AP PAAD Disease Free Survival
AQ PCGP Overall Survival
AR PCGP Disease Free Survival
Low CCL14 TPM
High CCL14 TPM
Percent survival
Months
U: Logrank p=0.17 HR(high)=0.35 p(HR)=0.19 n(high)=32 n(low)=32
V: Logrank p=0.75 HR(high)=1.2 p(HR)=0.75 n(high)=32 n(low)=32
W: Logrank p=0.46 HR(high)=0.89 p(HR)=0.47 n(high)=258 n(low)=258
X: Logrank p=0.47 HR(high)=0.87 p(HR)=0.47 n(high)=258 n(low)=258
Y: Logrank p=0.26 HR(high)=0.71 p(HR)=0.26 n(high)=141 n(low)=141
Z: Logrank p=0.98 HR(high)=0.99 p(HR)=0.98 n(high)=141 n(low)=141
AA: Logrank p=0.26 HR(high)=0.73 p(HR)=0.26 n(high)=53 n(low)=53
AB: Logrank p=1 HR(high)=1 p(HR)=NaN n(high)=53 n(low)=53
AC: Logrank p=0.0096 HR(high)=0.63 p(HR)=0.01 n(high)=257 n(low)=257
AD: Logrank p=0.2 HR(high)=0.82 p(HR)=0.2 n(high)=257 n(low)=257
AE: Logrank p=1e-04 HR(high)=0.5 p(HR)=0.00014 n(high)=182 n(low)=182
AF: Logrank p=0.02 HR(high)=0.7 p(HR)=0.021 n(high)=182 n(low)=182
AG: Logrank p=0.013 HR(high)=0.69 p(HR)=0.014 n(high)=239 n(low)=239
AH: Logrank p=0.085 HR(high)=0.77 p(HR)=0.085 n(high)=239 n(low)=239
AI: Logrank p=0.25 HR(high)=1.2 p(HR)=0.24 n(high)=241 n(low)=241
AJ: Logrank p=0.32 HR(high)=0.84 p(HR)=0.32 n(high)=241 n(low)=241
AK: Logrank p=0.22 HR(high)=1.3 p(HR)=0.22 n(high)=41 n(low)=41
AL: Logrank p=0.093 HR(high)=0.62 p(HR)=0.094 n(high)=41 n(low)=41
AM: Logrank p=0.1 HR(high)=1.2 p(HR)=0.11 n(high)=212 n(low)=212
AN: Logrank p=0.36 HR(high)=1.1 p(HR)=0.36 n(high)=212 n(low)=212
AO: Logrank p=0.68 HR(high)=0.92 p(HR)=0.68 n(high)=89 n(low)=89
AP: Logrank p=0.17 HR(high)=0.73 p(HR)=0.17 n(high)=89 n(low)=89
AQ: Logrank p=0.074 HR(high)=0.18 p(HR)=0.11 n(high)=91 n(low)=91
AR: Logrank p=0.55 HR(high)=0.75 p(HR)=0.55 n(high)=91 n(low)=91

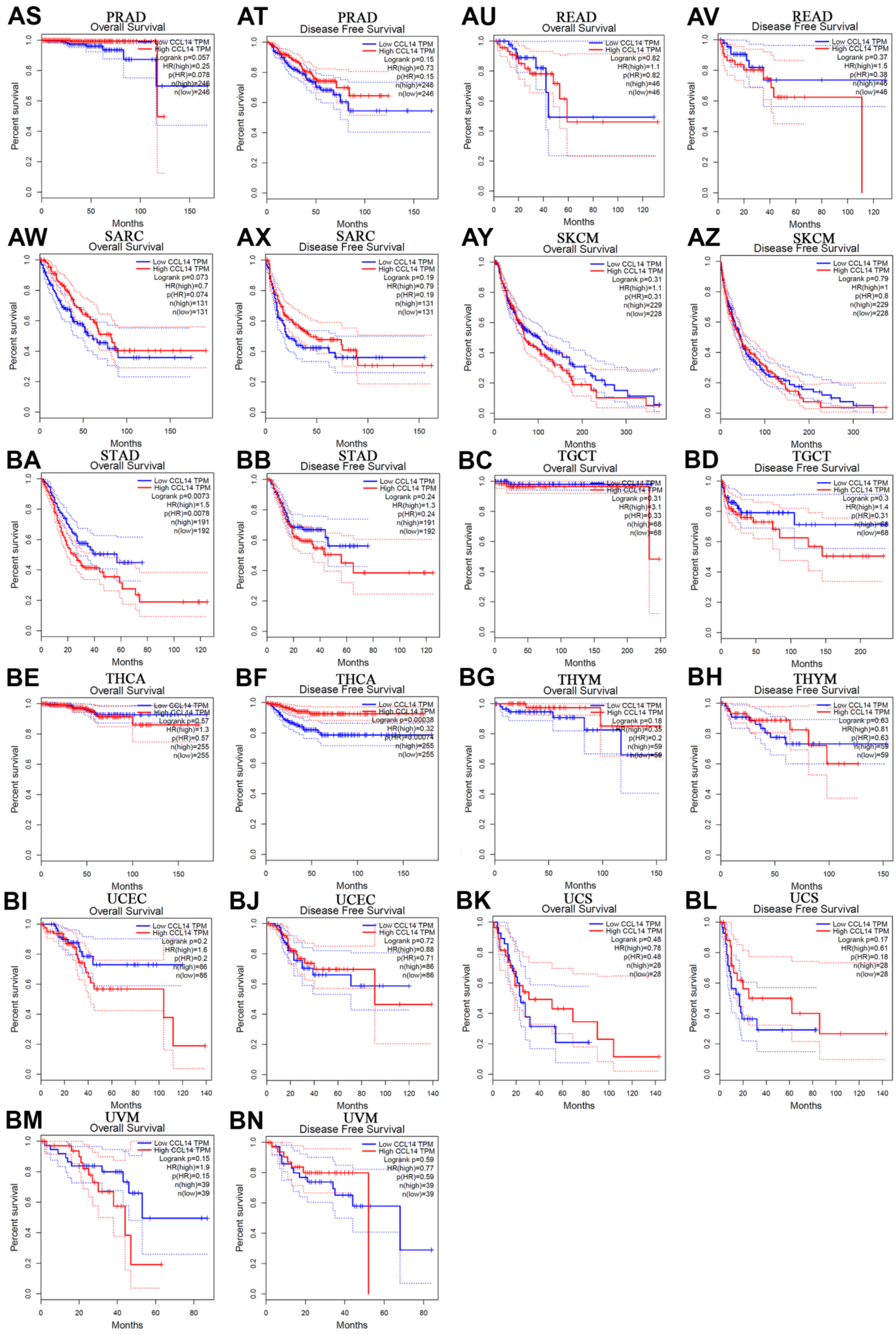
AS
PRAD
Overall Survival
Low CCL14 TPM
High CCL14 TPM
Logrank p=0.057
HR(high)=0.25
p(HR)=0.078
n(high)=246
n(low)=246
AT
PRAD
Disease Free Survival
Low CCL14 TPM
High CCL14 TPM
Logrank p=0.15
HR(high)=0.73
p(HR)=0.15
n(high)=246
n(low)=246
AU
READ
Overall Survival
Low CCL14 TPM
High CCL14 TPM
Logrank p=0.82
HR(high)=1.1
p(HR)=0.82
n(high)=46
n(low)=46
AV
READ
Disease Free Survival
Low CCL14 TPM
High CCL14 TPM
Logrank p=0.37
HR(high)=1.5
p(HR)=0.38
n(high)=46
n(low)=46
AW
SARC
Overall Survival
Low CCL14 TPM
High CCL14 TPM
Logrank p=0.073
HR(high)=0.7
p(HR)=0.074
n(high)=131
n(low)=131
AX
SARC
Disease Free Survival
Low CCL14 TPM
High CCL14 TPM
Logrank p=0.19
HR(high)=0.79
p(HR)=0.19
n(high)=131
n(low)=131
AY
SKCM
Overall Survival
Low CCL14 TPM
High CCL14 TPM
Logrank p=0.31
HR(high)=1.1
p(HR)=0.31
n(high)=229
n(low)=228
AZ
SKCM
Disease Free Survival
Low CCL14 TPM
High CCL14 TPM
Logrank p=0.79
HR(high)=1
p(HR)=0.8
n(high)=229
n(low)=228
BA
STAD
Overall Survival
Low CCL14 TPM
High CCL14 TPM
Logrank p=0.0073
HR(high)=1.5
p(HR)=0.0078
n(high)=191
n(low)=192
BB
STAD
Disease Free Survival
Low CCL14 TPM
High CCL14 TPM
Logrank p=0.24
HR(high)=1.3
p(HR)=0.24
n(high)=191
n(low)=192
BC
TGCT
Overall Survival
Low CCL14 TPM
High CCL14 TPM
Logrank p=0.31
HR(high)=3.1
p(HR)=0.33
n(high)=68
n(low)=68
BD
TGCT
Disease Free Survival
Low CCL14 TPM
High CCL14 TPM
Logrank p=0.3
HR(high)=1.4
p(HR)=0.31
n(high)=68
n(low)=68
BE
THCA
Overall Survival
Low CCL14 TPM
High CCL14 TPM
Logrank p=0.57
HR(high)=1.3
p(HR)=0.57
n(high)=255
n(low)=255
BF
THCA
Disease Free Survival
Low CCL14 TPM
High CCL14 TPM
Logrank p=0.00038
HR(high)=0.32
p(HR)=0.00074
n(high)=255
n(low)=255
BG
THYM
Overall Survival
Low CCL14 TPM
High CCL14 TPM
Logrank p=0.18
HR(high)=0.35
p(HR)=0.2
n(high)=59
n(low)=59
BH
THYM
Disease Free Survival
Low CCL14 TPM
High CCL14 TPM
Logrank p=0.63
HR(high)=0.81
p(HR)=0.63
n(high)=59
n(low)=59
BI
UCEC
Overall Survival
Low CCL14 TPM
High CCL14 TPM
Logrank p=0.2
HR(high)=1.6
p(HR)=0.2
n(high)=86
n(low)=86
BJ
UCEC
Disease Free Survival
Low CCL14 TPM
High CCL14 TPM
Logrank p=0.72
HR(high)=0.88
p(HR)=0.71
n(high)=86
n(low)=86
BK
UCS
Overall Survival
Low CCL14 TPM
High CCL14 TPM
Logrank p=0.48
HR(high)=0.78
p(HR)=0.48
n(high)=28
n(low)=28
BL
UCS
Disease Free Survival
Low CCL14 TPM
High CCL14 TPM
Logrank p=0.17
HR(high)=0.61
p(HR)=0.18
n(high)=28
n(low)=28
BM
UVM
Overall Survival
Low CCL14 TPM
High CCL14 TPM
Logrank p=0.15
HR(high)=1.9
p(HR)=0.15
n(high)=39
n(low)=39
BN
UVM
Disease Free Survival
Low CCL14 TPM
High CCL14 TPM
Logrank p=0.59
HR(high)=0.77
p(HR)=0.59
n(high)=39
n(low)=39
Percent survival
Months

Supplementary Figure 1. Correlation of CCL14 expression with prognostic values in diverse types of cancer in the GEPIA databases. Overall survival and disease free curves comparing the high and low expression of CCL14 in Adrenocortical carcinoma (ACC) (**A**–**B**), Bladder Urothelial Carcinoma (BLCA) (**C**–**D**), Breast invasive carcinoma (BRCA) (**E**–**F**), Cholangiocarcinoma (CHOL) (**G**–**H**), Colon adenocarcinoma (COAD) (**I**–**J**), Cervical squamous cell carcinoma and endocervical adenocarcinoma (CESC) (**K**–**L**), Lymphoid Neoplasm Diffuse Large B-cell Lymphoma (DLBC) (**M**–**N**), Esophageal carcinoma (ESCA) (**O**–**P**), Glioblastoma multiforme (GBM) (**Q**–**R**) ,Head and Neck squamous cell carcinoma (HNSC) (**S**–**T**), Kidney Chromophobe (KICH) (**U**–**V**), Kidney renal clear cell carcinoma (KIRC) (**W**–**X**), Kidney renal papillary cell carcinoma (KIRP) (**Y**–**Z**), Acute Myeloid Leukemia (LAML) (**AA**–**AB**), Brain Lower Grade Glioma (LGG) (**AC**–**AD**), Liver hepatocellular carcinoma (LIHC(HCC)) (**AE**–**AF**),Lung adenocarcinoma (LUAD) (**AG**–**AH**), Lung squamous cell carcinoma (LUSC) (**AI**–**AJ**), Mesothelioma (MESO) (**AK**–**AL**), Ovarian serous cystadenocarcinoma (OV) (**AM**–**AN**), Pancreatic adenocarcinoma (PAAD) (**AO**–**AP**), Pheochromocytoma and Paraganglioma (PCPG) (**AQ**–**AR**), Prostate adenocarcinoma (PRAD) (**AS**–**AT**)), Rectum adenocarcinoma (READ) (**AU**–**AV**), Sarcoma (SARC) (**AW**–**AX**), Skin Cutaneous Melanoma (SKCM) (**AY**–**AZ**), Stomach adenocarcinoma (STAD) (**BA**–**BB**), Testicular Germ Cell Tumors (TGCT) (**BC**–**BD**), Thyroid carcinoma (THCA) (**BE**–**BF**), Thymoma (THYM) (**BG**–**BH**), Uterine Corpus Endometrial Carcinoma (UCEC) (**BI**–**BJ**), Uterine Carcinosarcoma (UCS) (**BK**–**BL**), Uveal Melanoma (UVM) (**BM**–**BN**).

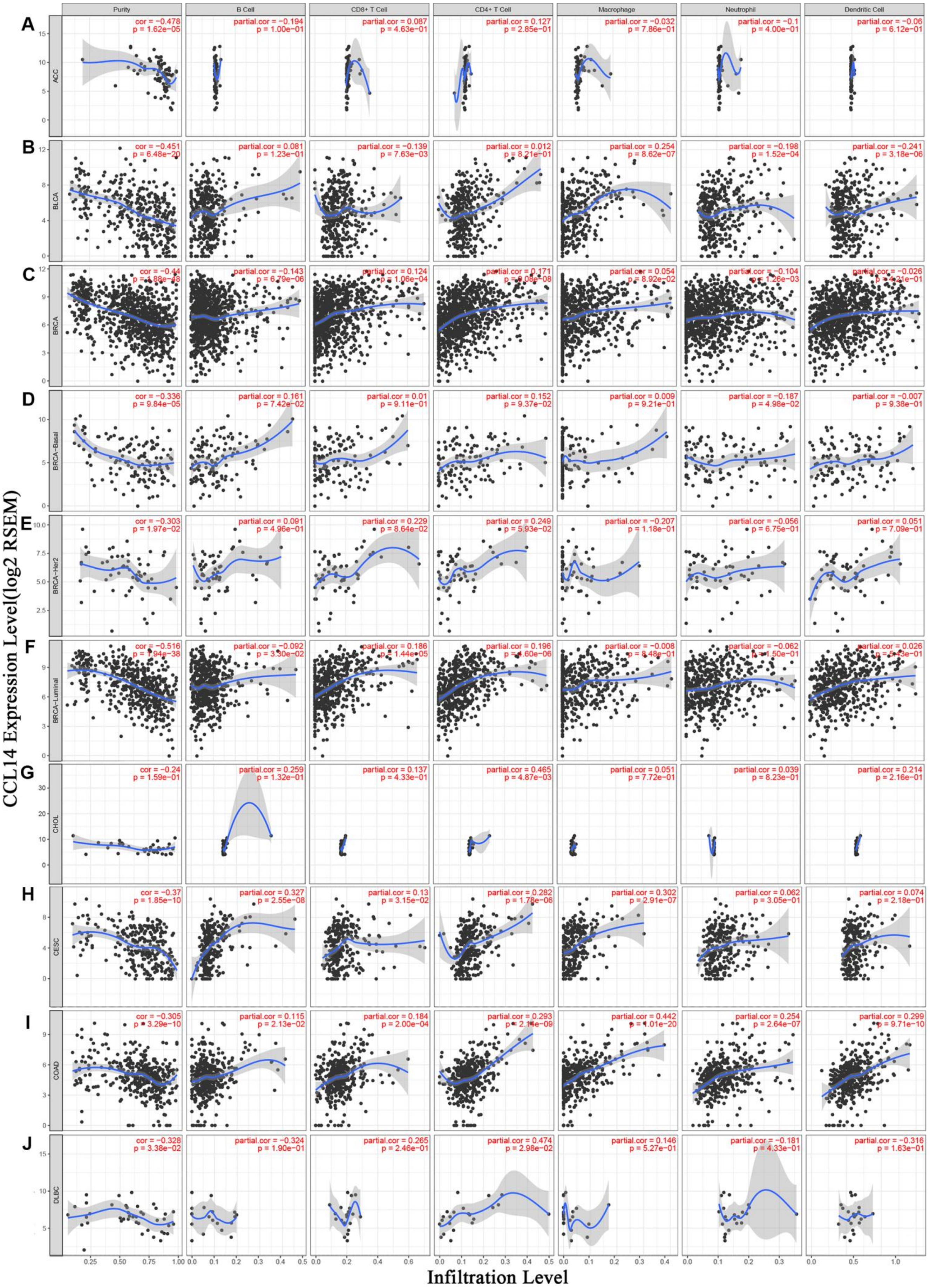

Purity
B Cell
CD8+ T Cell
CD4+ T Cell
Macrophage
Neutrophil
Dendritic Cell
A
B
C
D
E
F
G
H
I
J
ACC
BLCA
BRCA
BRCA−Basal
BRCA−Her2
BRCA−Luminal
CHOL
CESC
COAD
DLBC
CCL14 Expression Level(log2 RSEM)
Infiltration Level

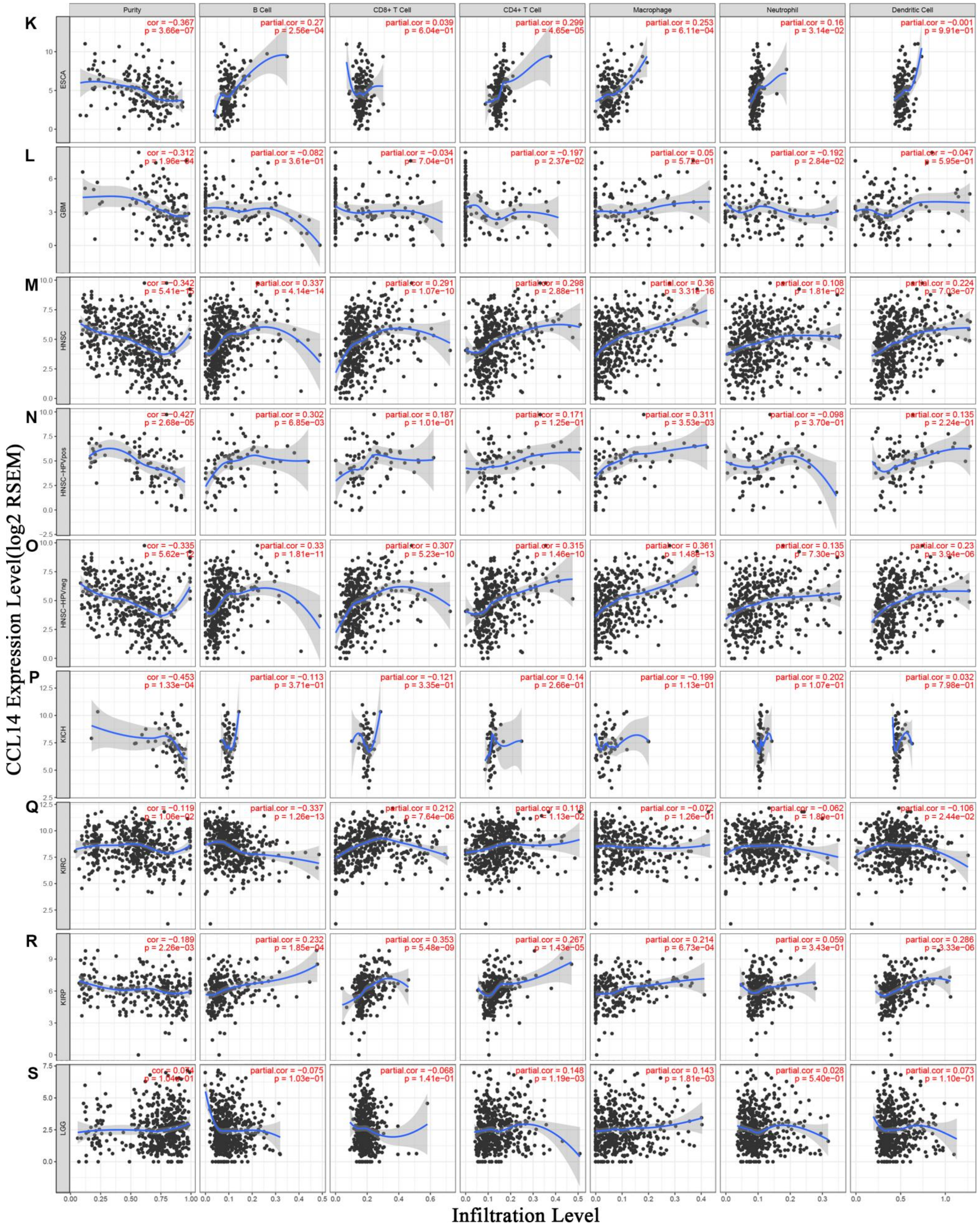

Purity
B Cell
CD8+ T Cell
CD4+ T Cell
Macrophage
Neutrophil
Dendritic Cell
K
ESCA
cor = −0.367 p = 3.66e-07
partial.cor = 0.27 p = 2.56e-04
partial.cor = 0.039 p = 6.04e-01
partial.cor = 0.299 p = 4.65e-05
partial.cor = 0.253 p = 6.11e-04
partial.cor = 0.16 p = 3.14e-02
partial.cor = −0.001 p = 9.91e-01
L
GBM
cor = −0.312 p = 1.96e-04
partial.cor = −0.082 p = 3.61e-01
partial.cor = −0.034 p = 7.04e-01
partial.cor = −0.197 p = 2.37e-02
partial.cor = 0.05
partial.cor = −0.192 p = 2.84e-02
partial.cor = −0.047 p = 5.95e-01
M
HNSC
cor = −0.342
partial.cor = 0.337 p = 4.14e-14
partial.cor = 0.291 p = 1.07e-10
partial.cor = 0.298 p = 2.88e-11
partial.cor = 0.36 p = 3.31e-16
partial.cor = 0.108 p = 1.81e-02
partial.cor = 0.224 p = 7.03e-07
N
HNSC−HPVpos
cor = −0.427 p = 2.68e-05
partial.cor = 0.302 p = 6.85e-03
partial.cor = 0.187 p = 1.01e-01
partial.cor = 0.171 p = 1.25e-01
partial.cor = 0.311 p = 3.53e-03
partial.cor = −0.098 p = 3.70e-01
partial.cor = 0.135 p = 2.24e-01
O
HNSC−HPVneg
cor = −0.335 p = 5.62e-12
partial.cor = 0.33 p = 1.81e-11
partial.cor = 0.307 p = 5.23e-10
partial.cor = 0.315 p = 1.46e-10
partial.cor = 0.361 p = 1.48e-13
partial.cor = 0.135 p = 7.30e-03
partial.cor = 0.23 p = 3.94e-06
P
KICH
cor = −0.453 p = 1.33e-04
partial.cor = −0.113 p = 3.71e-01
partial.cor = −0.121 p = 3.35e-01
partial.cor = 0.14 p = 2.66e-01
partial.cor = −0.199 p = 1.13e-01
partial.cor = 0.202 p = 1.07e-01
partial.cor = 0.032 p = 7.98e-01
Q
KIRC
cor = −0.119 p = 1.06e-02
partial.cor = −0.337 p = 1.26e-13
partial.cor = 0.212 p = 7.64e-06
partial.cor = 0.118 p = 1.13e-02
partial.cor = −0.072 p = 1.26e-01
partial.cor = −0.062 p = 1.89e-01
partial.cor = −0.106 p = 2.44e-02
R
KIRP
cor = −0.189 p = 2.26e-03
partial.cor = 0.232 p = 1.85e-04
partial.cor = 0.353 p = 5.48e-09
partial.cor = 0.267 p = 1.43e-05
partial.cor = 0.214 p = 6.73e-04
partial.cor = 0.059 p = 3.43e-01
partial.cor = 0.286 p = 3.33e-06
S
LGG
p = 1.04e-01
partial.cor = −0.075 p = 1.03e-01
partial.cor = −0.068 p = 1.41e-01
partial.cor = 0.148 p = 1.19e-03
partial.cor = 0.143 p = 1.81e-03
partial.cor = 0.028 p = 5.40e-01
partial.cor = 0.073 p = 1.10e-01
CCL14 Expression Level(log2 RSEM)
Infiltration Level

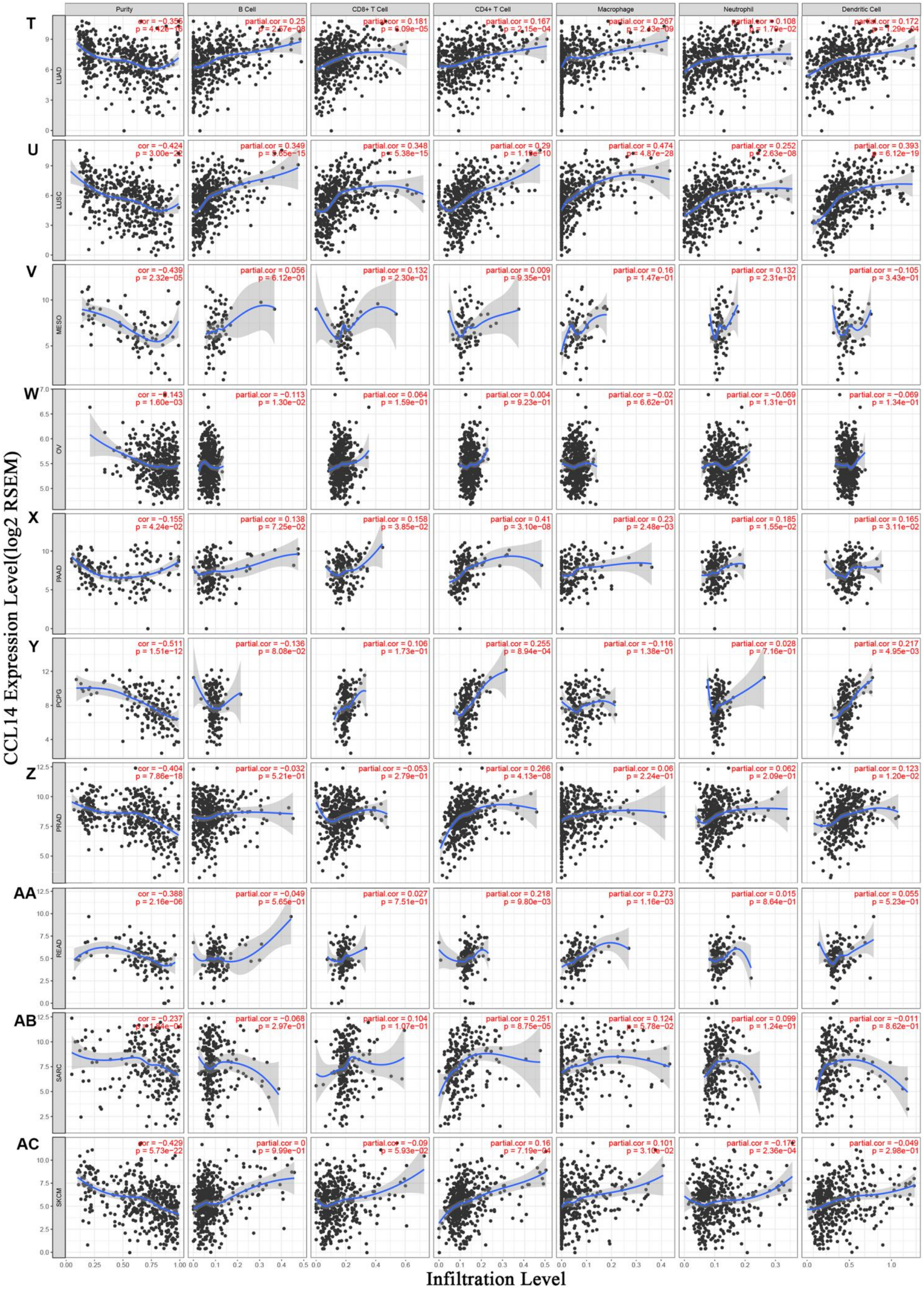
Purity
B Cell
CD8+ T Cell
CD4+ T Cell
Macrophage
Neutrophil
Dendritic Cell
T
LUAD
partial.cor = 0.25 p = 2.67e-08
partial.cor = 0.181 p = 6.09e-05
partial.cor = 0.167 p = 2.15e-04
partial.cor = 0.267 p = 2.43e-09
partial.cor = 0.108 p = 1.79e-02
partial.cor = 0.172 p = 1.29e-04
U
LUSC
cor = −0.424 p = 3.00e-22
partial.cor = 0.349 p = 5.65e-15
partial.cor = 0.348 p = 5.38e-15
partial.cor = 0.29 p = 1.19e-10
partial.cor = 0.474 p = 4.87e-28
partial.cor = 0.252 p = 2.63e-08
partial.cor = 0.393 p = 6.12e-19
V
MESO
cor = −0.439 p = 2.32e-05
partial.cor = 0.056 p = 6.12e-01
partial.cor = 0.132 p = 2.30e-01
partial.cor = 0.009 p = 9.35e-01
partial.cor = 0.16 p = 1.47e-01
partial.cor = 0.132 p = 2.31e-01
partial.cor = −0.105 p = 3.43e-01
W
OV
cor = −0.143 p = 1.60e-03
partial.cor = −0.113 p = 1.30e-02
partial.cor = 0.064 p = 1.59e-01
partial.cor = 0.004 p = 9.23e-01
partial.cor = −0.02 p = 6.62e-01
partial.cor = −0.069 p = 1.31e-01
partial.cor = −0.069 p = 1.34e-01
X
PAAD
cor = −0.155 p = 4.24e-02
partial.cor = 0.138 p = 7.25e-02
partial.cor = 0.158 p = 3.85e-02
partial.cor = 0.41 p = 3.10e-08
partial.cor = 0.23 p = 2.48e-03
partial.cor = 0.185 p = 1.55e-02
partial.cor = 0.165 p = 3.11e-02
Y
PCPG
cor = −0.511 p = 1.51e-12
partial.cor = −0.136 p = 8.08e-02
partial.cor = 0.106 p = 1.73e-01
partial.cor = 0.255 p = 8.94e-04
partial.cor = −0.116 p = 1.38e-01
partial.cor = 0.028 p = 7.16e-01
partial.cor = 0.217 p = 4.95e-03
Z
PRAD
cor = −0.404 p = 7.86e-18
partial.cor = −0.032 p = 5.21e-01
partial.cor = −0.053 p = 2.79e-01
partial.cor = 0.266 p = 4.13e-08
partial.cor = 0.06 p = 2.24e-01
partial.cor = 0.062 p = 2.09e-01
partial.cor = 0.123 p = 1.20e-02
AA
READ
cor = −0.388 p = 2.16e-06
partial.cor = −0.049 p = 5.65e-01
partial.cor = 0.027 p = 7.51e-01
partial.cor = 0.218 p = 9.80e-03
partial.cor = 0.273 p = 1.16e-03
partial.cor = 0.015 p = 8.64e-01
partial.cor = 0.055 p = 5.23e-01
AB
SARC
cor = −0.237
partial.cor = −0.068 p = 2.97e-01
partial.cor = 0.104 p = 1.07e-01
partial.cor = 0.251 p = 8.75e-05
partial.cor = 0.124 p = 5.78e-02
partial.cor = 0.099 p = 1.24e-01
partial.cor = −0.011 p = 8.62e-01
AC
SKCM
cor = −0.429 p = 5.73e-22
partial.cor = 0 p = 9.99e-01
partial.cor = −0.09 p = 5.93e-02
partial.cor = 0.16 p = 7.19e-04
partial.cor = 0.101 p = 3.10e-02
partial.cor = −0.172 p = 2.36e-04
partial.cor = −0.049 p = 2.98e-01
CCL14 Expression Level(log2 RSEM)
Infiltration Level

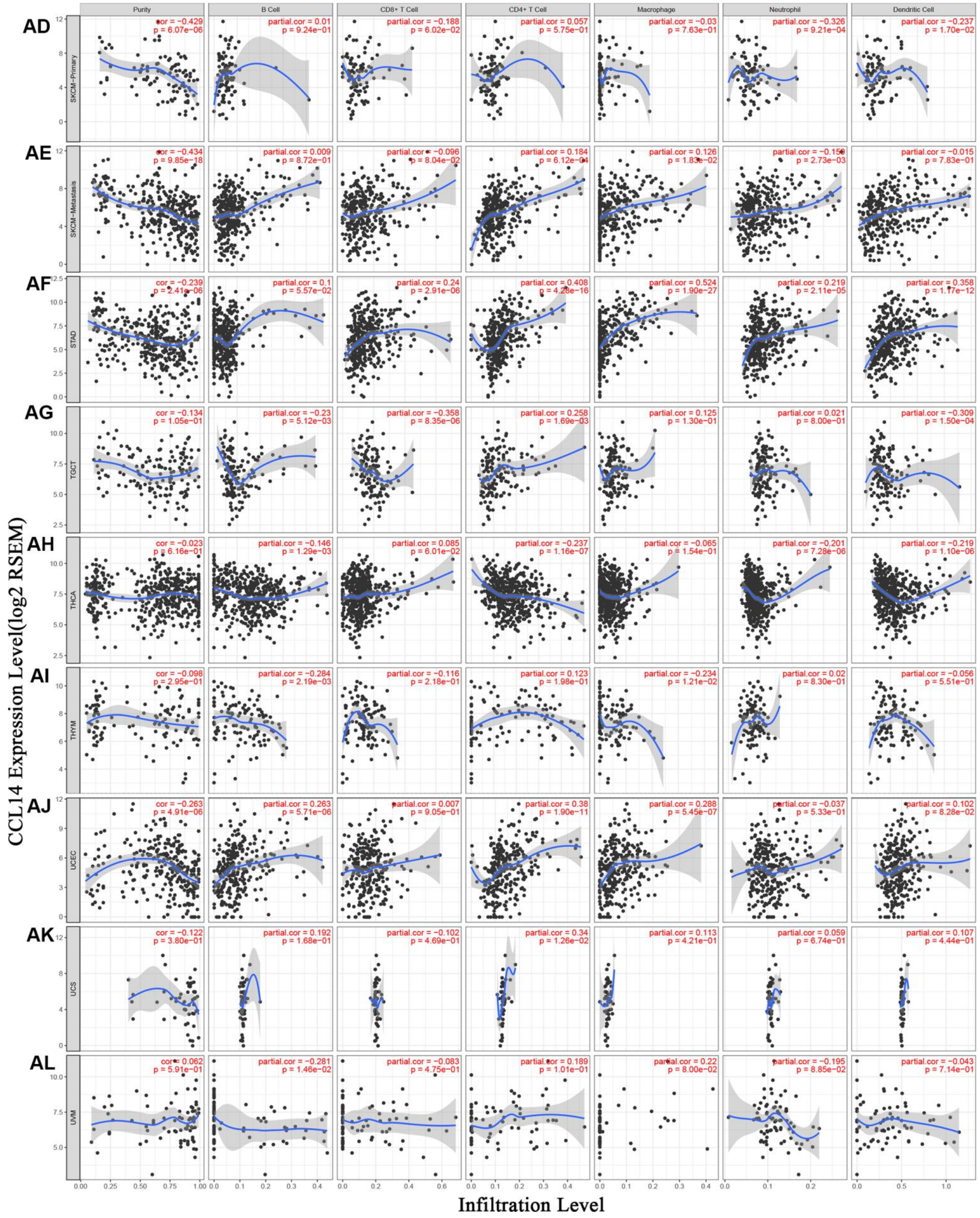

Supplementary Figure 2. Correlation of CCL14 expression with immune infiltration levels in diverse types of cancer in the TIMER database. Adrenocortical carcinoma (ACC) (**A**), Bladder Urothelial Carcinoma (BLCA) (**B**), Breast invasive carcinoma (BRCA)

(**C**), Breast invasive carcinoma-Basal (BRCA-Basal) (**D**), Breast invasive carcinoma-Her2 (BRCA-Her2) (**E**), Breast invasive carcinoma-Luminal (BRCA- Luminal) (**F**), Cholangiocarcinoma (CHOL)(**G**), Cervical squamous cell carcinoma and endocervical adenocarcinoma (CESC) (**H**), Colon adenocarcinoma (COAD) (**I**), Lymphoid Neoplasm Diffuse Large B-cell Lymphoma (DLBC) (**J**), Esophageal carcinoma (ESCA) (**K**), Glioblastoma multiforme (GBM)(**L**) ,Head and Neck squamous cell carcinoma (HNSC) (**M**), Head and Neck squamous cell carcinoma- HPVpos (HNSC-HPVpos) (**N**) , Head and Neck squamous cell carcinoma-HPVneg (HNSC-HPVneg) (**O**), Kidney Chromophobe (KICH) (**P**), Kidney renal clear cell carcinoma (KIRC) (**Q**), Kidney renal papillary cell carcinoma (KIRP) (**R**), Brain Lower Grade Glioma (LGG) (**S**), Lung adenocarcinoma (LUAD) (**T**), Lung squamous cell carcinoma (LUSC) (**U**), Mesothelioma (MESO) (**V**), Ovarian serous cystadenocarcinoma (OV) (**W**), Pancreatic adenocarcinoma (PAAD) (**X**), Pheochromocytoma and Paraganglioma (PCPG) (**Y**), Prostate adenocarcinoma (PRAD) (**Z**), Rectum adenocarcinoma (READ) (**AA**), Sarcoma (SARC) (**AB**), Skin Cutaneous Melanoma (SKCM) (**AC**), Skin Cutaneous Melanoma-Primary (SKCM-Primary) (**AD**), Skin Cutaneous Melanoma-Metastasis (SKCM- Metastasis) (**AE**), Stomach adenocarcinoma (STAD) (**AF**), Testicular Germ Cell Tumors (TGCT) (**AG**), Thyroid carcinoma (THCA) (**AH**), Thymoma (THYM) (**AI**), Uterine Corpus Endometrial Carcinoma (UCEC) (**AJ**), Uterine Carcinosarcoma (UCS) (**AK**), Uveal Melanoma (UVM) (**AL**).
