# Supplementary material for: CCL14 is a prognostic biomarker and correlates with immune infiltrates in hepatocellular carcinoma
Source: Aging (Albany NY). 2020 Jan 12;12(1):784–807. doi: 10.18632/aging.102656 (PMC6977663; doi:10.18632/aging.102656)
Supplement: Supplementary Table 1 [file aging-12-102656-s001..pdf]

SUPPLEMENTARY TABLES

Supplementary Table 1. CCL14 expression in cancers verus normal tissue in oncomine database.

Cancer	Cancer type	P-value	Fold change	Rank (%)	Sample	Reference (PMID)
Bladder	Superficial Bladder Cancer	1.04E-14	-2.67	2%	194	20421545
	Infiltrating Bladder Urothelial Carcinoma	3.08E-12	-2.64	1%	130	20421545
Brain and CNS	Classic Medulloblastoma	6.81E-06	4.89	6%	50	11807556
Breast	Ductal Breast Carcinoma	8.14E-05	-7.126	3%	69	11553815
Cervical	Cervical Cancer	3.75E-08	-2.651	2%	42	17510386
Colorectal	Colon Adenoma	4.90E-16	-2.895	1%	57	18171984
	Colorectal Carcinoma	6.48E-20	-3.738	1%	82	20143136
	Colorectal Adenocarcinoma	7.22E-14	-3.246	1%	69	20957034
	Colorectal Carcinoma	1.40E-11	-2.63	3%	60	20957034
	Colon Adenocarcinoma	1.55E-08	-2.028	4%	91	17640062
	Rectosigmoid Adenocarcinoma	3.69E-06	-2.116	2%	15	17615082
	Rectal Mucinous Adenocarcinoma	2.07E-06	-2.723	4%	28	TCGA
Esophageal	Barrett's Esophagus	2.87E-06	2.912	3%	43	16449976
Gastric	Gastric Intestinal Type Adenocarcinoma	5.93E-05	-2.091	5%	57	19081245
Head and neck	Floor of the Mouth Carcinoma	5.23E-07	-2.323	1%	27	17510386
	Oral Cavity Squamous Cell Carcinoma	7.52E-14	-2.524	2%	79	21853135
	Salivary Gland Adenoid Cystic Carcinoma	8.20E-05	-3.127	6%	22	12368205
	Head and Neck Squamous Cell Carcinoma	1.88E-10	-3.424	4%	54	14729608
	Tongue Squamous Cell Carcinoma	4.69E-05	-2.818	7%	57	19138406
Kidney	Papillary Renal Cell Carcinoma	6.11E-07	-6.74	2%	24	19445733
Liver	Hepatocellular Carcinoma	5.12E-74	-3.643	1%	445	21159642
Lung	Small Cell Lung Carcinoma	4.92E-06	-8.044	2%	23	11707567
	Lung Carcinoid Tumor	5.05E-08	-10.696	5%	37	11707567
	Large Cell Lung Carcinoma	2.06E-14	-6.549	2%	84	20421987
	Squamous Cell Lung Carcinoma	1.17E-13	-4.594	6%	92	20421987
	Lung Adenocarcinoma	2.96E-12	-3.424	4%	246	22080568
Lymphoma	Follicular Lymphoma	2.56E-22	5.311	2%	58	19412164
	Activated B-Cell-Like Diffuse Large B-Cell Lymphoma	5.72E-09	3.017	5%	37	19412164
	Diffuse Large B-Cell Lymphoma	2.99E-12	3.546	7%	64	19412164
Melanoma	Benign Melanocytic Skin Nevus	4.69E-07	-4.809	1%	25	16243793
	Cutaneous Melanoma	3.85E-13	-15.472	1%	52	16243793
Ovarian	Ovarian Serous Adenocarcinoma	1.36E-16	-178.012	1%	42	19486012
	Ovarian Serous Cystadenocarcinoma	6.00E-06	-11.856	3%	594	TCGA
Sarcoma	Uterine Corpus Leiomyosarcoma	5.43E-05	-17.273	1%	13	15101043
	Myxofibrosarcoma	4.67E-15	-6.67	1%	40	20601955
	Pleomorphic Liposarcoma	1.10E-11	-4.7	1%	32	20601955
	Leiomyosarcoma	4.63E-13	-4.899	1%	35	20601955
	Dedifferentiated Liposarcoma	4.65E-13	-3.998	1%	55	20601955
	Myxoid/Round Cell Liposarcoma	2.88E-09	-2.977	2%	29	20601955
	Fibrosarcoma	1.76E-05	-7.174	1%	22	15994966
	Malignant Fibrous Histiocytoma	7.11E-05	-6.442	2%	24	15994966
Others	Vulvar Intraepithelial Neoplasia	9.38E-06	-4.368	1%	19	17471573
	Malignant Fibrous Histiocytoma	7.11E-05	-6.442	2%	24	15994966
	Pleural Malignant Mesothelioma	2.12E-05	-2.442	3%	49	15920167
	Uterine Corpus Leiomyoma	9.86E-05	-2.305	4%	77	19622772
